# ADC Conjugation Strategies: From Technological Evolution to a Practical Selection Framework

**DOI:** 10.3390/pharmaceutics18070852

**Published:** 2026-07-13

**Authors:** Sanggil Kim, Se-Ra Lee, Myung-Ho Sohn, Kyungeun Lee, Shin-Woong Kim, Hojin Yoo, Hyeonju Jeong, Donghyun Oh, Jinwoong Chang, Jeong-ah Yun, So-Young Choi

**Affiliations:** Team of Biologics Optimization, Division of Biologics Discovery, New Drug Development Center, Osong Medical Innovation Foundation (KBIOHealth), Cheongju 28160, Chungbuk, Republic of Korea; ryon2174@kbiohealth.kr (S.K.); srlee@kbiohealth.kr (S.-R.L.); sohnho2@kbiohealth.kr (M.-H.S.); gkak0829@kbiohealth.kr (K.L.); shinwg.kim@kbiohealth.kr (S.-W.K.); yohojin@kbiohealth.kr (H.Y.); hyeonju0602@kbiohealth.kr (H.J.); tomato@kbiohealth.kr (D.O.); johnwoong@kbiohealth.kr (J.C.); yjj50236@kbiohealth.kr (J.-a.Y.)

**Keywords:** antibody–drug conjugates (ADCs), conjugation strategy, site-specific conjugation, site-selective conjugation, drug-to-antibody ratio (DAR)

## Abstract

Antibody–drug conjugates (ADCs) have received renewed attention in oncology following the clinical and commercial success of agents such as trastuzumab deruxtecan (Enhertu^®^), which underscored the clinical importance of coordinated optimization of the antibody, linker, payload, and conjugation strategy. Among these parameters, conjugation strategy strongly influences drug-to-antibody ratio (DAR) distribution, pharmacokinetics, manufacturability, and safety. Over the past two decades, ADC conjugation technologies have evolved from first-generation stochastic modification approaches to second-generation site-specific engineering strategies and, more recently, to third-generation site-selective platforms applicable to native antibodies. First-generation approaches remain the most clinically validated and have the strongest regulatory precedent despite their intrinsic heterogeneity. Second-generation approaches improve positional control and DAR homogeneity, but often increase demands on antibody engineering, expression, and process complexity. Third-generation approaches aim to preserve controlled conjugation while reducing sequence-engineering requirements, yet remain limited by a restricted number of accessible sites and less extensive clinical and regulatory experience. In this review, we summarize the evolution of ADC conjugation technologies, highlight representative clinical and translational examples, and discuss the major trade-offs of each platform. We also propose a practical selection framework based on payload properties, required DAR control, antibody stability, manufacturability, and regulatory considerations, with relevance to next-generation ADCs including dual-payload and bispecific formats. Overall, conjugation strategy should be selected according to the scientific, manufacturing, and regulatory requirements of each ADC program rather than on the basis of a presumed technological hierarchy.

## 1. Introduction

Antibody–drug conjugates (ADCs) represent an important class of targeted therapeutics that combine the high specificity of monoclonal antibodies (mAbs) with the potent cytotoxicity of small-molecule drugs [1,2]. By covalently linking a cytotoxic payload to an antibody through a chemical linker, ADCs can selectively deliver therapeutic agents to tumor cells while sparing normal tissues, thereby expanding the therapeutic window. Structurally, an ADC is composed of three essential elements—the antibody, linker, and payload—each of which contributes to overall efficacy, safety, and translational performance [1].

The clinical success of trastuzumab deruxtecan (T-DXd; Enhertu^®^), a human epidermal growth factor receptor 2 (HER2)-directed ADC carrying a topoisomerase I inhibitor payload (deruxtecan, Dxd) through a cleavable tetrapeptide-based linker, has renewed broad interest in the ADC field. Its activity in HER2-low tumors highlighted how target biology, linker design, payload properties, and conjugation methodology can collectively influence clinical performance [3]. This progress has stimulated continued innovation across ADC design, from molecular engineering to manufacturing.

Among these design parameters, the conjugation strategy—defining how and where the payload is attached to the antibody—is an important determinant of drug-to-antibody ratio (DAR) distribution, pharmacokinetic behavior, therapeutic index, and product consistency [4,5]. Over the past two decades, ADC conjugation technologies have expanded from first-generation stochastic modification approaches to second-generation site-specific engineering strategies and, more recently, to third-generation site-selective platforms that can be applied to native antibodies [6,7]. Each class of conjugation technology addresses a different balance among molecular precision, antibody compatibility, manufacturability, and development complexity.

Importantly, the conjugation strategy can also influence key biological properties of ADCs. The conjugation site and chemical linkage may modulate antibody–receptor interactions, internalization efficiency, and intracellular trafficking, ultimately affecting payload release and pharmacological potency. Moreover, antigen biology—such as internalization dynamics, receptor recycling, or shedding—must align with conjugation design to achieve efficient delivery. Conjugation near the Fc region can additionally alter FcγR or neonatal Fc receptor (FcRn) interactions, influencing immune effector function, serum half-life, and immunogenicity [8]. Thus, rational ADC design requires integrated consideration of both chemical precision and biological compatibility.

In parallel, regulatory authorities have increasingly emphasized quality attributes such as DAR distribution, aggregation control, and reproducibility of conjugation processes [9]. These expectations have strengthened interest in controlled conjugation approaches, particularly when tighter product definition is required for complex payloads or emerging ADC formats. At the same time, clinically established stochastic platforms remain highly relevant because of their regulatory familiarity, scalable manufacturing workflows, and extensive translational precedent. Accordingly, the practical value of a conjugation strategy depends not only on the degree of molecular control it offers, but also on how well it fits the chemical, biological, manufacturing, and regulatory context of a given ADC program.

In this review, we do not simply retrace the chemical evolution of ADC conjugation technologies. Instead, we examine how stochastic, site-specific, and site-selective platforms address different development problems and where each is most appropriately applied. We compare these strategies not only in terms of molecular precision, but also with respect to payload potency and hydrophobicity, required DAR control, antibody structural stability, compatibility with native antibody assets, manufacturability, and regulatory precedent [4,5,8,9]. On this basis, we propose a practical selection framework intended to guide conjugation strategy choice in real development settings and to clarify when the added complexity of second- or third-generation platforms is justified, particularly for increasingly demanding formats such as dual-payload and bispecific ADCs.

For this narrative review, relevant literature was identified through targeted searches of PubMed, Web of Science, and Scopus using combinations of terms related to antibody–drug conjugates, conjugation chemistry, site-specific and site-selective conjugation, glycan remodeling, disulfide rebridging, linker design, drug-to-antibody ratio, manufacturing, and chemistry, manufacturing, and controls (CMC). Peer-reviewed primary studies, relevant reviews, regulatory documents, and registered clinical-trial records were included when they provided information on conjugation mechanisms, product characteristics, translational development, or CMC considerations. Clinical-trial status and regulatory information were verified using ClinicalTrials.gov, Food and Drug Administration (FDA) prescribing information, Drugs@FDA records, and FDA approval announcements. Duplicate publications, studies unrelated to therapeutic ADCs, and sources lacking sufficient methodological or product-specific information were excluded, and reference lists of relevant publications were screened for additional primary studies. The literature, regulatory, and clinical-trial information was updated through 2 July 2026.

## 2. Evolution and Design Considerations of ADC Conjugation Technologies

Before discussing individual platforms, it should be noted that the boundaries between first-, second-, and third-generation conjugation technologies are not absolute and may vary across the literature. In this review, we adopt a pragmatic, development-oriented classification intended to support comparative evaluation. Here, the term native antibodies refers to wild-type, non-engineered antibodies used without sequence modification.

Within this framework, first-generation approaches refer to stochastic conjugation methods that modify endogenous residues naturally present on antibodies, most notably lysines and interchain cysteines. Second-generation approaches refer to site-specific strategies that depend on antibody engineering or engineered recognition motifs. Rather than representing a universally accepted mechanistic class, the term “third-generation” is used here to describe emerging site-selective conjugation strategies that enable controlled payload attachment on native or minimally engineered antibodies. Accordingly, selected disulfide rebridging platforms are discussed within this category when implemented on native antibodies, with classification based primarily on native antibody compatibility and translational or CMC considerations rather than historical nomenclature alone. Overall, these categories are intended as a development-oriented framework for comparative evaluation of ADC conjugation strategies.

### 2.1. First-Generation: Stochastic Conjugation

First-generation ADCs relied on stochastic (non-site-specific) conjugation methods that target endogenous amino acid residues naturally present on antibodies, most notably lysines and interchain cysteines [9]. These conjugation chemistries are straightforward and compatible with unmodified antibodies, enabling rapid development and manufacturing.

#### 2.1.1. Lysine Conjugation

**Figure 1:**
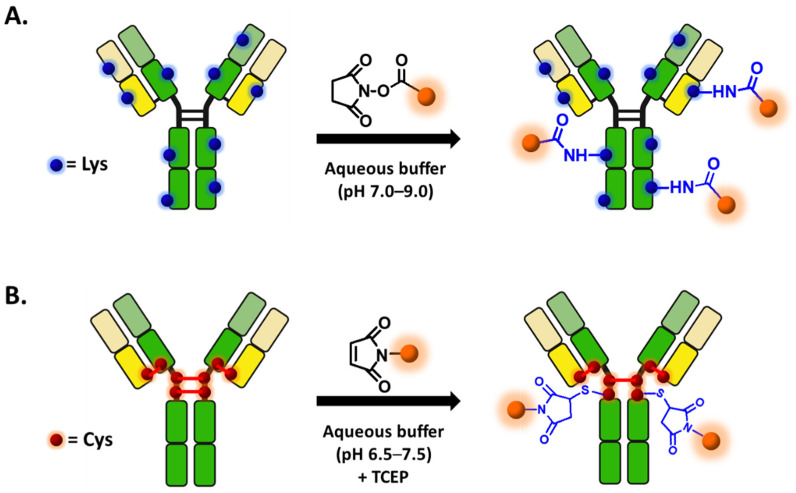
Schematic illustration of lysine- and cysteine-based stochastic conjugation in first-generation ADCs. (**A**) Lysine conjugation via amine-reactive chemistry generates heterogeneous ADC populations with variable DAR. (**B**) Cysteine conjugation following partial disulfide reduction enables thiol-specific coupling with improved but still variable DAR. The schematic is simplified and may not capture the full extent of product heterogeneity or process-related impurities.

Amine-reactive chemistry, typically using *N*-hydroxysuccinimide (NHS) esters or sulfo-NHS esters, has been widely used to functionalize lysine ε-amino groups [10]. A typical IgG1 molecule contains approximately 80–90 lysine residues, with around 30–40 being solvent-accessible and chemically reactive [1]. Because these lysine residues are stochastically distributed across both heavy and light chains and differ in solvent exposure, local microenvironment, and intrinsic reactivity, lysine conjugation generates a highly heterogeneous mixture of ADC species with diverse conjugation sites and a broad distribution of drug-to-antibody ratios (DARs). Although clinically developed lysine-conjugated ADCs are commonly controlled to achieve a defined average DAR, the actual DAR distribution and average DAR can vary substantially depending on the antibody scaffold, linker–payload properties, and conjugation conditions [11,12]. The lack of positional control leads to substantial heterogeneity in the final ADC product (Figure 1A).

Several early ADCs developed using lysine conjugation received FDA approval. Gemtuzumab ozogamicin (Mylotarg^®^), a CD33-directed ADC carrying the DNA-cleaving calicheamicin payload through an acid-labile hydrazone linker, was first approved in 2000 for acute myeloid leukemia (AML) and re-approved in 2017 [13]. Trastuzumab emtansine (T-DM1; Kadcyla^®^) is a HER2-directed ADC composed of trastuzumab linked to the microtubule inhibitor DM1 through a non-cleavable Succinimidyl 4-(*N*-maleimidomethyl) cyclohexane-1-carboxylate (SMCC)-derived linker [14]. Inotuzumab ozogamicin (Besponsa^®^) is a CD22-directed ADC that delivers a calicheamicin payload through an acid-labile hydrazone linker [15]. However, lysine-based conjugation can interfere with antigen binding and introduces batch-to-batch variability, complicating pharmacokinetic prediction and quality control [16].

#### 2.1.2. Cysteine Conjugation

In contrast, cysteine conjugation involves the partial reduction of the four interchain disulfide bonds in IgG1 antibodies to generate reactive thiol groups for coupling. Maleimide chemistry is most commonly used, forming thioether linkages with accessible cysteine residues (Figure 1B). This approach generally results in products with DARs of 2, 4, 6, or 8 and improved homogeneity compared to lysine conjugation [17].

Although cysteine-based conjugation reduces heterogeneity, concerns remain about structural stability. Disrupting disulfide bonds can compromise antibody integrity, and the maleimide–thiol linkage is subject to retro-Michael addition, potentially leading to premature payload release and off-target toxicity [18].

#### 2.1.3. Practical Considerations and Limitations of Stochastic Conjugation

Both lysine- and cysteine-based stochastic conjugation methods generate heterogeneous ADC populations. Even at the same average DAR, positional isomers may exhibit distinct pharmacokinetic behavior, toxicity, and aggregation tendencies. Broad DAR distributions and limited positional control can complicate analytical characterization and may narrow the therapeutic index, particularly when highly potent or hydrophobic payloads are used [19]. At the same time, the practical relevance of stochastic conjugation remains substantial. As of early 2025, more than 1800 ADC development programs had been catalogued globally, with approximately 23% (424 candidates) having entered various stages of clinical development. A significant proportion of these clinical-stage ADCs still rely on lysine- or cysteine-based stochastic conjugation platforms, underscoring the continued practicality, flexibility, and commercial viability of these first-generation strategies [20]. In addition, all FDA-approved ADCs listed in Table 1 employ either lysine- or cysteine-based stochastic conjugation approaches (Table 1), further highlighting their strong clinical and regulatory precedent [21,22,23,24]. For cysteine-based conjugation in particular, partial reduction of interchain disulfide bonds may affect antibody structural stability depending on the intrinsic resilience of the antibody scaffold, and the resulting conjugates can be further influenced by payload hydrophobicity and DAR. In addition, classical maleimide–thiol linkages have been associated with limited stability due to retro-Michael deconjugation [18]. Accordingly, the value of stochastic conjugation lies not in maximal molecular precision, but in its balance of clinical maturity, manufacturability, and developmental practicality despite its inherent molecular heterogeneity [25].

### 2.2. Second-Generation: Site-Specific Conjugation via Antibody Engineering

Second-generation conjugation technologies were developed to achieve precise, site-specific attachment of cytotoxic payloads and tighter control over conjugation site and DAR than first-generation stochastic approaches. By reducing positional heterogeneity, these methods can improve product consistency and, in some settings, pharmacokinetic behavior or therapeutic index [4,40]. Mechanistically, these technologies can be grouped into engineered cysteine conjugation, unnatural amino acid (UAA) incorporation, and enzymatic or chemoenzymatic tagging. Selected representative programs are summarized in Table 2, whereas the complete program-level listing is provided in Supplementary Table S1.

#### 2.2.1. Engineered Cysteine Conjugation

Engineered cysteine conjugation introduces cysteine residues at predefined antibody positions by site-directed mutagenesis, enabling selective thiol coupling while generally preserving antigen binding and overall structure [51]. Compared with stochastic conjugation, it provides tighter control over DAR and positional distribution, but site choice remains critical because additional sulfhydryl groups may promote disulfide scrambling or unintended intermolecular interactions [40,52].

**Figure 2:**
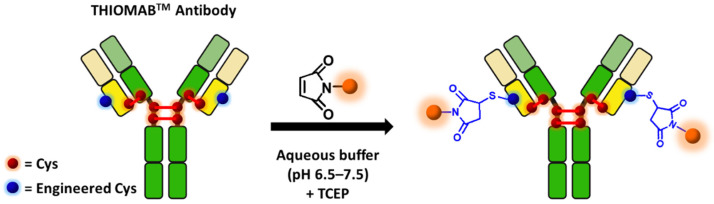
Schematic illustration of engineered cysteine–based site-specific conjugation in second-generation ADCs using the THIOMAB platform. Engineered cysteine residues enable site-specific thiol coupling with defined DAR. The schematic is simplified and may not capture the full extent of product heterogeneity or process-related impurities.

***THIOMAB.*** THIOMAB is the best-established example of engineered cysteine–based thiol conjugation. Non-critical residues such as HC-A114C and LC-V110C, along with several alternative heavy- and light-chain sites, have been used to create defined conjugation handles with limited impact on Fc or antigen-binding functions (Figure 2) [4,6,51,53]. THIOMAB-based ADCs are generally designed to achieve a defined DAR of 2 with high product homogeneity [6]. DCDS0780A and MEDI2228 have been evaluated in completed Phase I studies [42,54], whereas HDP-101 remains in Phase I/II evaluation and the Phase I study of BYON3521 has been completed [43,55]. Thus, engineered cysteine conjugation remains a clinically relevant route to homogeneous ADCs, although local structural context and conjugate stability still require careful optimization.

#### 2.2.2. Unnatural Amino Acid (UAA) Incorporation

UAA conjugation uses genetic code expansion to incorporate noncanonical residues bearing orthogonal reactive handles, such as ketones or azides, at predefined amber codons [56]. These handles enable bioorthogonal ligation under mild conditions and can generate highly homogeneous ADCs with defined DAR, but reduced expression yield, truncated chain formation, and potential immunogenicity remain important practical considerations [57,58].

**Figure 3:**
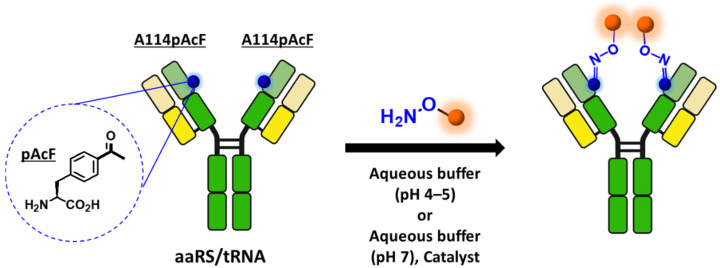
Schematic illustration of site-specific conjugation via unnatural amino acid (UAA) incorporation in second-generation ADCs. Incorporation of an unnatural amino acid (e.g., *p*-acetylphenylalanine) at predefined sites enables bioorthogonal conjugation, yielding homogeneous ADCs with a defined DAR. The schematic is simplified and may not capture the full extent of product heterogeneity or process-related impurities.

***EuCODE.*** EuCODE enables incorporation of p-acetylphenylalanine (pAcF) into antibodies expressed in Chinese hamster ovary (CHO) cells, followed by conjugation of aminooxy-modified payloads through oxime ligation, typically yielding ADCs with a DAR of approximately 2, high homogeneity, and scalable manufacturing potential (Figure 3) [59,60,61,62]. ARX788 is the most clinically advanced EuCODE example and is supported by preclinical studies, published Phase I findings, and later-phase clinical evaluation in HER2-positive breast cancer [45,63,64,65,66,67].

***XpressCF+***. XpressCF+ enables unnatural amino acid incorporation using a cell-free protein synthesis system, facilitating rapid construct design and controlled folding conditions while supporting current good manufacturing practice (cGMP)-oriented scale-up [7,46,58,68]. STRO-002, generated by site-specific UAA incorporation to achieve DAR 4, demonstrated preclinical activity in ovarian cancer models and was evaluated in a completed Phase I study [47]. Overall, UAA-based platforms offer very high molecular precision, but they also impose specialized manufacturing requirements.

#### 2.2.3. Enzymatic and Chemoenzymatic Tagging

Enzymatic and chemoenzymatic tagging strategies use engineered recognition motifs that are selectively processed by enzymes, enabling traceless or regioselective payload installation under mild conditions [69,70]. Their key advantages are high site selectivity and compatibility with diverse payload chemistries, whereas their main limitations are the need for sequence engineering and possible constraints related to enzyme turnover or tag accessibility.

**Figure 4:**
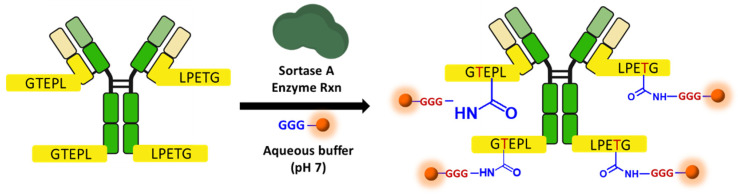
Schematic illustration of Sortase A–mediated site-specific conjugation in second-generation ADCs using the SMAC platform. Sortase A enables site-specific transpeptidation of LPXTG-engineered antibodies with GGG-modified payloads, yielding homogeneous ADCs with a defined DAR. The schematic is simplified and may not capture the full extent of product heterogeneity or process-related impurities.

***SMAC*.** SMAC is a Sortase A–mediated transpeptidation platform that uses LPXTG motifs introduced at antibody termini to enable site-specific ligation of GGG-modified payloads, typically yielding conjugates with a DAR of approximately 4 while preserving antibody structure and activity (Figure 4) [71,72]. NBE-002 demonstrated antitumor activity in preclinical ovarian cancer models; however, its Phase I/II study was terminated, and the available evidence remains limited to preclinical and early clinical development [48,73].

**Figure 5:**
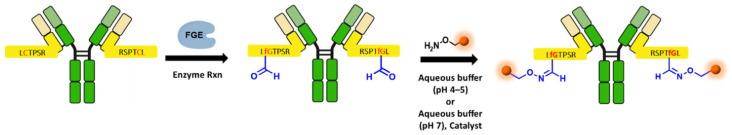
Schematic illustration of FGE-mediated aldehyde-tag–based site-specific conjugation in second-generation ADCs using the SMARTag platform. FGE converts an engineered CxPxR motif into formylglycine, enabling site-specific conjugation and yielding homogeneous ADCs with a defined DAR. The schematic is simplified and may not capture the full extent of product heterogeneity or process-related impurities.

***SMARTag***. SMARTag is a formylglycine-generating enzyme (FGE)-mediated aldehyde-tag conjugation platform that converts an engineered CxPxR sequence into formylglycine, enabling chemoselective ligation to aminooxy-functionalized payloads (Figure 5) [74,75]. This strategy typically yields conjugates with a DAR of approximately 1.8 and high plasma stability. In a completed Phase I study, TRPH-222 showed preliminary tolerability and antitumor activity in patients with relapsed/refractory B-cell non-Hodgkin lymphoma [49,76].

**Figure 6:**
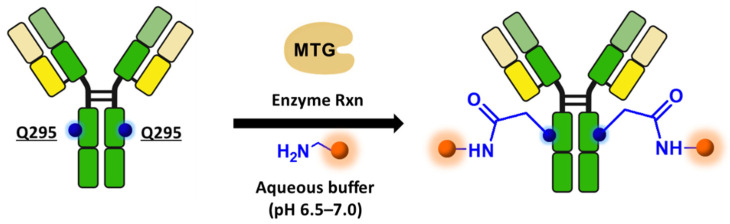
Schematic illustration of glutamine-directed enzymatic conjugation in second-generation ADCs using microbial transglutaminase (mTG). mTG catalyzes transamidation at engineered glutamine residues, yielding homogeneous ADCs with a defined DAR. The schematic is simplified and may not capture the full extent of product heterogeneity or process-related impurities.

***Microbial Transglutaminase (mTG).*** mTG is a glutamine-directed enzymatic conjugation platform that catalyzes transamidation at engineered glutamine residues such as Q295, enabling homogeneous ADC production under mild conditions (Figure 6) [77,78,79,80]. DP303c, a HER2-targeting ADC generated using this approach, demonstrated antitumor activity in preclinical models and preliminary safety and efficacy signals in a first-in-human clinical study [45,50,81].

***ConjuALL.*** ConjuALL is a CAAX/farnesyl transferase–mediated conjugation platform that uses a C-terminal CAAX motif to enable installation of a unique chemical handle for subsequent payload attachment, thereby preserving antibody function while enabling defined DAR and high homogeneity [45]. LCB84 is a representative Trop-2-directed program undergoing early-phase clinical evaluation, although mature clinical outcome data remain limited in the public domain [44].

Collectively, second-generation platforms provide tighter control over DAR and positional distribution than stochastic conjugation and have been associated in selected studies with improved product consistency and translational predictability. These benefits, however, must be weighed against the added burdens of antibody engineering, specialized expression systems, and process integration. Selected examples in Table 2 illustrate different levels of clinical maturity, including active early-phase programs, completed studies, and a terminated program. The complete program-level listing, including indications, sponsors, trial identifiers, and registry status, is provided in Supplementary Table S1. Registry status refers to the individual study and does not necessarily establish continuation or discontinuation of the overall development program. Preliminary early-phase findings are described as such, and inclusion in Table 2 or Supplementary Table S1 should not be interpreted as evidence of established clinical benefit.

### 2.3. Third-Generation: Site-Selective Conjugation on Native Antibodies

Third-generation conjugation strategies aim to achieve controlled payload attachment on native, non-engineered antibodies. Rather than introducing new sequence elements, these platforms exploit intrinsic antibody features such as Fc glycans, Fc-proximal lysines, or native disulfides (Table 3). This can be advantageous when established antibody assets are already available, because it may reduce the need for sequence redesign, cell-line redevelopment, and extensive process re-optimization. Selected representative programs are summarized in Table 3, whereas the complete program-level listing is provided in Supplementary Table S2.

#### 2.3.1. Fc Glycan Remodeling

Glycan remodeling technologies target the conserved N-linked glycosylation site at Asn297 in the Fc region. Native glycan structures are enzymatically trimmed and rebuilt to introduce chemical handles for site-selective conjugation.

**Figure 7:**
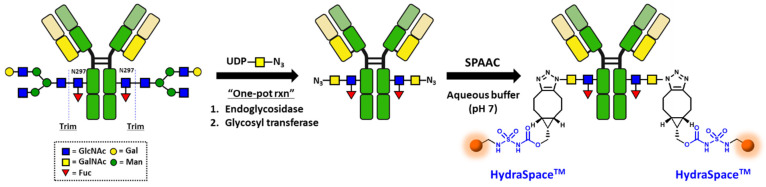
Schematic illustration of Fc glycan remodeling–based site-selective conjugation in third-generation ADCs using the GlycoConnect platform. Native Fc N-glycans at Asn297 are enzymatically trimmed and remodeled to introduce defined reactive handles, which are subsequently used for click chemistry-mediated linker–payload attachment. This approach enables site-selective conjugation on native antibodies without antibody sequence engineering, thereby improving conjugation-site control and DAR homogeneity while preserving the native antibody framework. The schematic is simplified and may not capture the full extent of product heterogeneity or process-related impurities.

***GlycoConnect*.** GlycoConnect is the best-established example of Fc glycan remodeling–based conjugation. It uses endoglycosidase-mediated trimming followed by glycosyltransferase-based remodeling to introduce defined reactive groups on the Fc glycan, enabling controlled conjugation under mild conditions without sequence engineering (Figure 7) [90,91]. In combination with HydraSpace linker technology, the platform can tune DAR and improve solubility or aggregation behavior [92,93,94,95,96]. In Phase I studies, IBI343 showed a manageable safety profile and preliminary antitumor activity in CLDN18.2-positive gastric or gastroesophageal junction and pancreatic cancer cohorts, supporting its subsequent progression into later-stage clinical evaluation [82,83,84].

#### 2.3.2. Proximity-Induced Conjugation

Proximity-induced conjugation platforms use Fc-binding peptides or proteins to position reactive groups near specific lysine residues, enabling site-selective modification of native antibodies while preserving the underlying sequence.

***AJICAP*.** AJICAP is an Fc-affinity peptide–directed lysine conjugation platform that selectively modifies defined Fc lysines, particularly Lys248 or Lys288, using cleavable peptide-mediated chemistry, enabling one-pot production of ADCs with a DAR of approximately 2 without redox treatment [97]. The platform has shown good conjugation efficiency at preparative scale, preserved FcRn binding, and favorable preclinical efficacy and tolerability in trastuzumab-based models [97,98].

**Figure 8:**
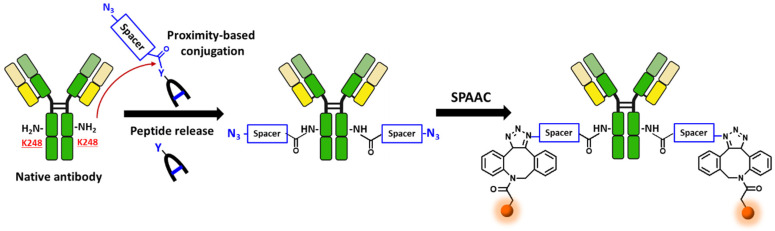
Schematic illustration of proximity-driven Lys248 acyl-transfer conjugation in third-generation ADCs using the AbClick platform. AbClick enables site-selective modification of Lys248 on native antibodies, yielding homogeneous ADCs with a defined DAR. The schematic is simplified and may not capture the full extent of product heterogeneity or process-related impurities.

***AbClick.*** AbClick is a proximity-driven Lys248 acyl-transfer conjugation platform that uses an Fc-binding peptide to enable site-selective modification of native antibodies, after which the peptide is removed in a traceless manner (Figure 8) [99]. The platform supports ADC formats with DAR 2 and DAR 4, including dual-payload installation, and preclinical studies have demonstrated antitumor activity for durvalumab–MMAE conjugates [100]. An emerging example is AT-211 (DA-3501), a CLDN18.2-targeting MMAE ADC developed using the AbClick^®^ platform for gastric, gastroesophageal junction, and pancreatic cancers. DA-3501 has entered first-in-human Phase I/IIa evaluation in patients with advanced gastric, gastroesophageal junction, and pancreatic ductal adenocarcinoma; however, mature clinical outcome data were not publicly available at the literature cut-off date [87].

**Figure 9:**
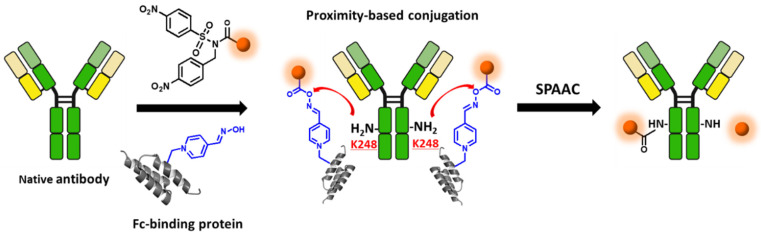
Schematic illustration of Fc-binding mediator–assisted traceless lysine conjugation in third-generation ADCs using the Z-M protein platform. The Z-M protein enables proximity-driven modification of Lys248 on native antibodies, yielding homogeneous ADCs with a DAR of approximately 2 following mediator dissociation. The schematic is simplified and may not capture the full extent of product heterogeneity or process-related impurities.

***Z-M Protein Platform.*** The Z-M protein platform is an Fc-binding mediator–assisted traceless lysine conjugation strategy that enables site-selective modification of native antibodies. The Z-M scaffold is engineered via genetic code expansion to incorporate *N*ε-azido-lysine (AzK), enabling proximity-driven acylation of Lys248 and installation of an azide handle for subsequent strain-promoted azide–alkyne cycloaddition (SPAAC)-based payload conjugation. Following conjugation, the mediator dissociates in a traceless manner, yielding homogeneous ADCs with a DAR of approximately 2 while maintaining HER2 and FcRn binding in trastuzumab-based constructs. (Figure 9) [101]. In preclinical evaluation, trastuzumab-(VC–PABC–MMAE)_2_ generated using this strategy showed enhanced in vitro cytotoxicity relative to T-DM1 under comparable conditions while retaining HER2 and FcRn binding [101].

#### 2.3.3. Disulfide Rebridging on Native Antibodies

Disulfide rebridging platforms are included here when they achieve controlled conjugation on native antibodies without sequence engineering. From a development perspective, their main attraction is the ability to improve homogeneity and linkage stability while retaining native antibody compatibility.

***WuXiDAR4.*** WuXiDAR4 is a hinge-selective native disulfide modification platform that uses Zn^2+^-mediated reduction followed by bifunctional linker insertion to enrich homogeneous ADCs with a DAR of approximately 4 while maintaining compatibility with native IgG1 manufacturing workflows [102,103]. Preclinical and platform reports suggest improved efficacy, acceptable tolerability, and cost of goods comparable to conventional stochastic conjugation, and several ADC programs using this approach have entered clinical development [6,102,103].

**Figure 10:**
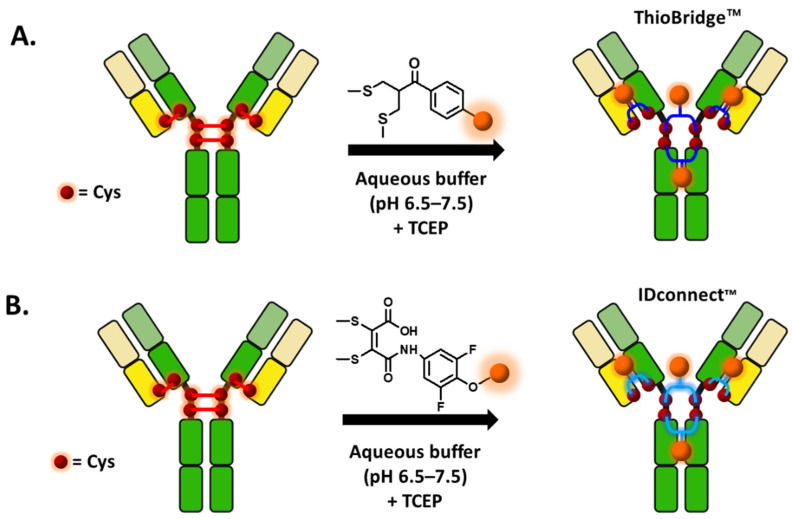
Schematic illustration of disulfide rebridging–based site-selective conjugation in third-generation ADCs. (**A**) ThioBridge employs a bis-sulfone scaffold to reconnect reduced interchain disulfide bonds, enabling controlled cysteine conjugation and yielding homogeneous ADCs with a defined DAR. (**B**) IDconnect utilizes a maleimide-based rebridging scaffold to achieve site-selective cysteine conjugation, yielding homogeneous ADCs with a defined DAR. The schematic is simplified and may not capture the full extent of product heterogeneity or process-related impurities.

***ThioBridge.*** ThioBridge is a bis-sulfone–based disulfide rebridging platform that reconnects reduced interchain disulfides to form stable bis-thioether linkages, thereby reducing retro-Michael liabilities associated with classical maleimide chemistry (Figure 10A) and enabling homogeneous ADCs with a DAR of approximately 4 [104,105,106]. ThioBridge conjugates have shown enhanced chemical stability under selected reducing conditions with minimal aggregation in preclinical evaluations [104]. MBRC-101 is undergoing early-phase clinical evaluation, with limited public clinical outcome data available at the literature cut-off date [88,107].

***IDconnect.*** IDconnect is a maleimide-based disulfide rebridging platform, part of Mabwell’s IDDC platform, that uses a proprietary scaffold substituted with a 4-mercaptobenzoyl-morpholine leaving group at the 3- and 4-positions (Figure 10B). This approach enables cross-linking of reduced cysteines in the Fab and hinge regions, generating highly homogeneous ADCs with a DAR of approximately 4 while suppressing deconjugation [108,109,110]. The nectin-4-targeting ADC 9MW2821 demonstrated strong preclinical activity and has advanced into Phase III evaluation, while the published clinical evidence remains primarily early-phase [89,110,111].

Taken together, third-generation platforms extend controlled conjugation to native antibodies and are especially relevant when preservation of an existing antibody sequence is a major development priority. Their remaining constraints include the limited number of accessible sites on native IgGs, reagent or process complexity, and comparatively limited clinical precedent for several platforms. Clinical programs based on these platforms differ substantially in maturity. IBI343 [82,83,84] and 9MW2821 [89] have advanced into Phase III evaluation, whereas AT-211 [87] and MBRC-101 [88] remain in early clinical development and mature public outcome data remain limited. MRG004A is supported by completed early-phase evaluation [85,86]. Selected examples are summarized in Table 3, and the complete program-level listing is provided in Supplementary Table S2. These examples illustrate the translational progress of the respective conjugation platforms and should not be interpreted as evidence of established clinical efficacy.

### 2.4. Influence of Linker Selection on ADC Properties

Linker design—including spacer composition, cleavage mechanism, self-immolative elements, and overall hydrophilicity—is a key determinant of ADC stability, safety, manufacturability, and therapeutic performance. An appropriate linker should remain stable during manufacturing and systemic circulation while enabling efficient intracellular payload release after target-mediated internalization. Cleavable linkers rely on acid-labile, reduction-sensitive, or protease-cleavable motifs, whereas non-cleavable linkers generally require lysosomal degradation of the antibody to generate payload-containing catabolites. The preferred release mechanism therefore depends on target biology, intracellular trafficking, and payload properties [112].

Linker stability in circulation strongly influences ADC tolerability and safety. Premature linker cleavage can increase systemic exposure to free payload and contribute to off-target toxicity, whereas inefficient intracellular release may reduce pharmacological activity. Cleavable linkers carrying membrane-permeable payloads may also produce a bystander effect, which can improve activity in heterogeneous tumors but may increase exposure of neighboring antigen-negative cells. Accordingly, the balance between extracellular stability and intracellular release efficiency directly influences payload delivery, antitumor activity, and the therapeutic index [112].

The physicochemical properties of the linker also affect manufacturing consistency and scalability. Hydrophobic linker–payload combinations can increase aggregation, reduce solubility, and complicate purification, particularly at higher DARs, whereas hydrophilic spacers can mitigate these effects and improve pharmacokinetic behavior or therapeutic index [19,92]. More complex linker structures may nevertheless require additional synthetic steps, tighter raw-material controls, and more extensive process characterization. Reaction robustness, impurity clearance, batch-to-batch consistency, and compatibility with scalable processes should therefore be considered during CMC development [113].

Accordingly, linker selection should be integrated with antigen internalization and trafficking, payload potency and membrane permeability, conjugation site, and target DAR. Rather than being considered in isolation, linker design should be treated as a cross-cutting variable that balances circulation stability, controlled intracellular release, safety, manufacturability, and therapeutic performance within each ADC program.

### 2.5. Comparative Perspective on ADC Conjugation Strategies: Toward a Practical Selection Framework

From a development perspective, conjugation strategy selection is driven by payload potency and hydrophobicity, the degree of DAR control required, the structural tolerance of the antibody scaffold, the importance of preserving the native antibody sequence, and the extent of CMC or regulatory burden that can be accommodated [4,5,8,19]. The comparative value of first-, second-, and third-generation platforms therefore depends on how they balance these variables.

**Figure 11:**
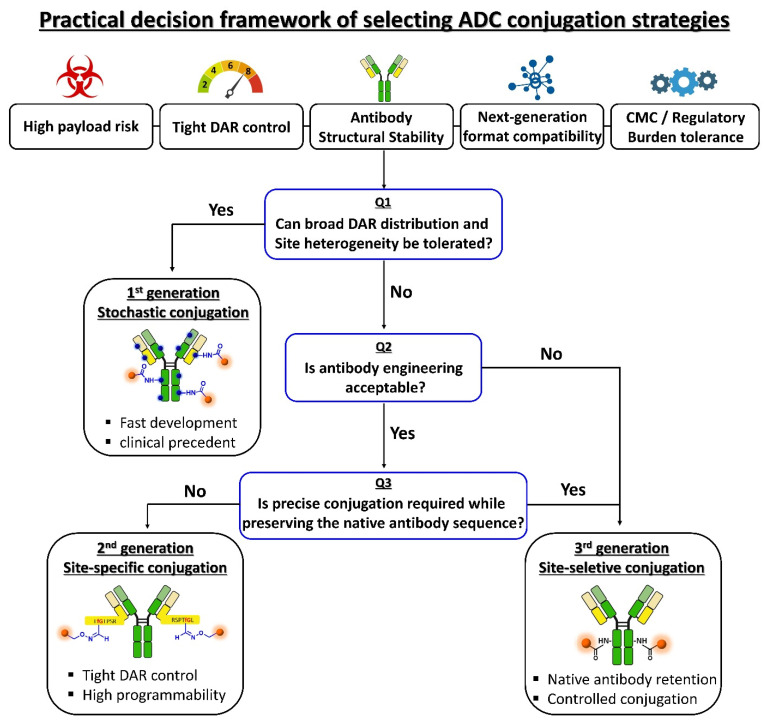
Practical decision framework for selecting ADC conjugation strategies. Stepwise selection is guided by payload risk, required DAR precision, antibody structural stability, compatibility with next-generation ADC formats, and CMC constraints. The framework first considers whether broad DAR distribution and site heterogeneity can be tolerated, followed by whether antibody engineering is acceptable and whether precise conjugation is required while preserving the native antibody sequence. The framework supports context-dependent selection of an appropriate conjugation strategy.

Based on these considerations, Figure 11 summarizes a stepwise decision framework that links payload-related risk, required DAR precision, antibody structural constraints, compatibility with next-generation ADC formats, and CMC feasibility to the selection of an appropriate conjugation strategy.

In this framework, high payload risk refers to the use of highly potent and/or hydrophobic payloads for which broad DAR distributions or positional heterogeneity may increase aggregation risk, alter pharmacokinetics, or narrow the therapeutic index [5,8,19]. When applying this criterion, linker stability and the hydrophobicity of the overall linker–payload construct should also be evaluated as interacting design factors, because they can further influence circulation stability, aggregation, pharmacokinetic behavior, and the therapeutic index [19,92,112]. Tight DAR control is required when individual DAR species materially influence pharmacokinetics, safety, or manufacturability, or when defined payload stoichiometry is needed, as in dual-payload ADCs. CMC burden tolerance reflects the extent to which a development program can accommodate antibody engineering, specialized reagents, multistep processing, and expanded analytical characterization [113]. These qualitative criteria should be considered together with antibody structural stability, format complexity, and regulatory precedent.

To illustrate the practical application of this framework, two representative use cases can be considered. First, an established native antibody combined with a moderate-risk payload and a rapid-development objective may favor a clinically established first-generation stochastic platform when broader DAR distribution and site heterogeneity are acceptable. Second, a hydrophobic or dual-payload ADC requiring defined payload stoichiometry may favor a second-generation site-specific strategy when antibody engineering is feasible. When preservation of the native antibody sequence is a priority, a third-generation site-selective approach may instead be considered. These examples are illustrative rather than prescriptive and should be interpreted in the context of the overall development program.

Within this framework, first-generation stochastic conjugation remains the most clinically validated and operationally established approach. All FDA-approved ADCs listed in Table 1 rely on lysine- or cysteine-based stochastic conjugation strategies, reflecting strong regulatory familiarity, established manufacturing workflows, and clear translational precedent [21,22,23,24]. However, these approaches inherently produce heterogeneous ADC populations. Although relatively narrower DAR distributions can be achieved through interchain cysteine conjugation, this strategy still depends on disulfide reduction, and both DAR and payload hydrophobicity can influence the resulting stability and developability of the conjugate. In addition, classical cysteine–maleimide conjugation has been associated with limited linker stability due to retro-Michael deconjugation [18]. Thus, the principal value of first-generation conjugation lies not in maximal molecular precision, but in its balance of clinical maturity, manufacturability, and developmental practicality.

Second-generation site-specific conjugation strategies were developed to achieve more homogeneous and structurally defined ADCs. By enabling precise payload attachment through engineered cysteine residues, unnatural amino acid incorporation, or enzymatic tagging, these approaches provide tighter control over DAR and positional distribution and, in selected studies and specific payload contexts, have been associated with improved plasma stability, pharmacokinetic consistency, or therapeutic performance relative to conventional stochastic formats [6,9,40]. However, these advantages are accompanied by additional development burdens, including sequence engineering, specialized incorporation systems, extra enzymatic processing steps, and, in some cases, reduced production yield or increased risk of perturbing antibody stability. Third-generation site-selective conjugation strategies seek to preserve the positional control advantages of more precise conjugation while reducing the engineering burden associated with second-generation platforms. By exploiting intrinsic structural features of native antibodies, these approaches may be particularly attractive when established antibody assets are already available. However, their available conjugation sites remain constrained by the limited structural features accessible on native antibodies. Except for selected glycan-remodeling and disulfide-rebridging platforms, most site-selective approaches remain at the preclinical or early clinical stage.

Taken together, these observations suggest that conjugation strategy selection should be approached in a stepwise manner. In practice, the initial decision is often shaped by whether the payload and antibody scaffold can tolerate the heterogeneity and structural consequences associated with less controlled conjugation formats. Subsequent considerations include the degree of DAR control required, the need to minimize positional heterogeneity, compatibility with more complex architectures such as dual-payload or bispecific ADCs, and practical development constraints including CMC feasibility, manufacturability, scalability, and regulatory familiarity. Under this framework, first-generation strategies remain highly competitive when rapid and practical development is prioritized, whereas second- and third-generation approaches become increasingly justified when greater molecular precision or programmability is required. Accordingly, strategy selection should align the required degree of molecular control with practical development constraints. Beyond molecular homogeneity, conjugation strategy can also influence developability-relevant attributes such as aggregation propensity, solubility, purification behavior, and storage stability. Accordingly, controlled conjugation may function as an upstream design variable that affects not only ADC structure but also downstream CMC development. Importantly, these effects should not be interpreted as consequences of DAR or conjugation site alone; linker architecture and payload physicochemical properties can further modify how conjugation heterogeneity translates into stability, pharmacokinetics, aggregation, and CMC risk [5,19,112,113]. The key practical considerations underlying conjugation strategy selection are summarized in Table 4.

## 3. Future Perspectives: Toward Multi-Payload and Multi-Specific ADCs

The rapid evolution of ADC conjugation technologies has not only enhanced product homogeneity and pharmacokinetic predictability but also expanded the structural possibilities of ADC design. As tumor heterogeneity, adaptive resistance, and antigen escape increasingly limit the durability of single-mechanism therapeutics, next-generation ADC development is moving toward advanced ADC architectures, including dual-payload ADCs and multi-specific targeting strategies.

### 3.1. Dual-Payload ADCs

**Figure 12:**
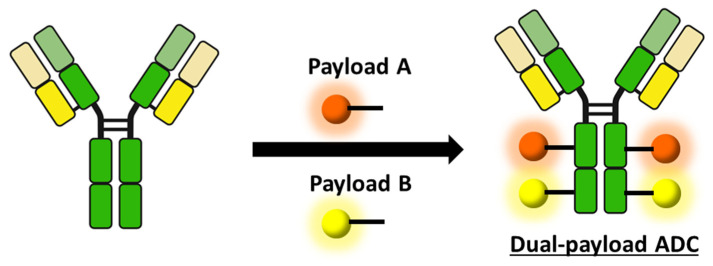
Schematic illustration of a dual-payload ADC. The antibody is conjugated to two distinct cytotoxic payloads, resulting in a dual-payload ADC with defined payload composition and DAR. The schematic is a simplified conceptual representation and does not depict all possible product variants, DAR species, positional isomers, or process-related impurities.

Dual-payload ADCs are designed to incorporate two distinct cytotoxic agents within a single antibody molecule, typically combining mechanistically complementary payloads such as a microtubule inhibitor and a DNA-damaging agent (Figure 12). This strategy aims to address intratumoral heterogeneity and reduce the likelihood of resistance by enabling simultaneous delivery of more than one cytotoxic mechanism within the same targeted construct [114,115].

In this setting, conjugation strategy becomes particularly important because dual-payload architectures impose greater demands on control over DAR, payload distribution, and compositional reproducibility. Broadly heterogeneous conjugation formats may complicate the definition of not only total DAR but also payload stoichiometry and positional distribution when two chemically distinct drugs are incorporated into the same antibody. This consideration is supported by homogeneous dual-payload ADC studies reported in the literature, in which controlled co-installation of distinct payloads on a single antibody improved antitumor activity in models of HER2 heterogeneity and drug resistance relative to matched single-payload formats or combination-style controls [116]. 

These findings illustrate how increasing architectural complexity can strengthen the practical justification for more controlled conjugation approaches. In this context, second- and third-generation strategies may become particularly relevant when dual-payload ADC development requires reproducible definition of both total DAR and payload composition, together with improved structural consistency across batches [116,117].

### 3.2. Multi-Specific and Bispecific ADCs

**Figure 13:**
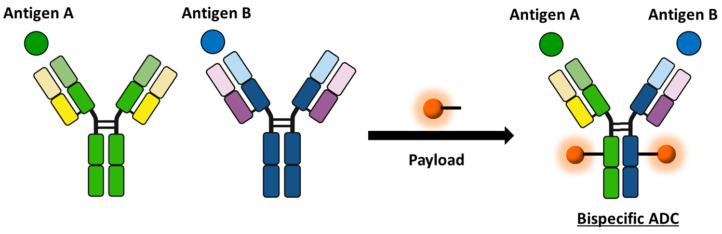
Schematic illustration of a bispecific ADC. The antibody is engineered to recognize two distinct target antigens, resulting in a bispecific ADC with dual-target specificity. The schematic is a simplified conceptual representation and does not depict all possible product variants, structural heterogeneity, positional isomers, or process-related impurities.

Beyond payload diversification, multi-specific ADCs including bispecific antibodies conjugated to cytotoxic drugs represent another promising direction in ADC design (Figure 13). By engaging two tumor-associated antigens, bispecific ADCs may improve target selectivity, enhance internalization in certain biological settings, and reduce the risk of antigen escape [118,119].

However, the increased structural complexity of bispecific formats introduces additional demands on conjugation strategy. In asymmetric or heterodimeric antibody architectures, payload distribution may need to be controlled without compromising antigen binding, chain pairing, or Fc-mediated properties. Under these conditions, conjugation approaches that provide tighter control over site, stoichiometry, and payload placement may offer practical advantages, particularly when modularity and reproducibility are required across more structurally complex ADC formats [120]. This translational relevance is underscored by BL-B01D1, a first-in-class EGFR × HER3 bispecific ADC carrying a topoisomerase I inhibitor payload, which showed preliminary antitumor activity with an acceptable safety profile in first-in-human phase I evaluation in patients with locally advanced or metastatic solid tumors [121].

Bispecific and multi-specific ADCs therefore represent an important use case in which the need for tighter control over conjugation site, stoichiometry, and payload placement may justify the use of second- or third-generation conjugation strategies.

## 4. Regulatory Considerations: CMC Relevance of Conjugation Strategy

As ADCs advance through clinical development and commercialization, conjugation strategy influences both the clinical pharmacology package and the CMC strategy. Although closely related, these areas address distinct development questions. Clinical pharmacology focuses on the exposure, disposition, and pharmacological effects of ADC-related analytes in patients, whereas CMC focuses on product definition, heterogeneity, stability, process robustness, and manufacturing consistency [9,113].

### 4.1. Clinical Pharmacology and Bioanalytical Considerations

Conjugation strategy shapes the bioanalytical package required to support ADC development. Clinical pharmacology assessment generally requires fit-for-purpose methods for total antibody, conjugated antibody or ADC, unconjugated payload, and relevant metabolites or catabolites, together with assessment of immunogenicity, including anti-drug antibody responses. Broad DAR distributions or insufficient conjugate stability can increase bioanalytical complexity because these analytes may exhibit distinct pharmacokinetic and safety profiles. Accordingly, dose- and exposure-response analyses should consider the relevant ADC-related analytes rather than relying solely on total antibody exposure or average DAR. Drug–drug interaction risk should also be evaluated when the released payload or its metabolites interact with drug-metabolizing enzymes or transporters [9,113].

### 4.2. CMC and Analytical Control of Conjugated Products

From a CMC perspective, conjugation-related critical quality attributes include DAR distribution, conjugation-site occupancy, aggregation, unconjugated payload, deconjugation, and process-related impurities. Average DAR alone may not fully capture the physicochemical and biological variability of an ADC product. Broad DAR distributions can affect batch consistency, dosing precision, safety, and comparability, whereas aggregation may increase immunogenicity risk and reduce bioavailability. Premature or nonspecific payload release may additionally increase off-target toxicity and narrow the therapeutic index. The principal regulatory and analytical implications of these attributes are summarized in Table 5.

These attributes generally require an orthogonal analytical control strategy. Hydrophobic interaction chromatography–high-performance liquid chromatography (HIC-HPLC), where informative for the product, can support DAR profiling and assessment of hydrophobic species. Intact or subunit mass spectrometry and liquid chromatography–mass spectrometry (LC–MS) peptide mapping can provide information on molecular mass, conjugation sites, and site occupancy, whereas size-exclusion chromatography (SEC) is commonly used to monitor aggregates and fragments. Assays for free payload and unconjugated linker–payload species are also important for controlling drug-related impurities. Stability-indicating studies should evaluate changes in DAR distribution, payload loss, aggregation, fragmentation, and biological activity, while residual reagent testing may be required for reducing agents, enzymes, affinity reagents, and other process-related materials [113].

First-generation stochastic conjugation generally increases the analytical burden associated with broad DAR distributions and positional heterogeneity. Second-generation site-specific approaches can reduce these sources of variability but may introduce additional controls related to antibody engineering, expression systems, or enzymatic processing. Third-generation site-selective approaches may improve product definition while preserving the native antibody sequence, although specialized reagents, platform maturity, and workflow robustness remain important considerations. Accordingly, tighter molecular control may facilitate product characterization and comparability but does not automatically reduce the overall regulatory or CMC burden. Its practical value depends on analytical capability, manufacturing robustness, scalability, and available regulatory precedent [5,53,113].

## 5. Conclusions

The comparative evidence reviewed here indicates that conjugation strategy should be selected according to the specific development problem it is intended to solve. Conjugation chemistry determines not only DAR distribution and product homogeneity, but also how effectively an ADC balances potency, stability, manufacturability, and translational risk.

From this perspective, first-generation stochastic conjugation remains well justified when speed, regulatory precedent, and manufacturing simplicity are prioritized, and when the antibody scaffold and payload can tolerate broader DAR distributions. Second-generation strategies become more compelling when precise DAR control, tighter positional definition, or greater programmability is needed, but these gains come with engineering and process burdens. Third-generation platforms are particularly attractive when developers wish to preserve a native antibody sequence while improving control over conjugation, although their broader clinical and regulatory experience is still developing.

As ADCs move toward dual-payload, bispecific, and other structurally demanding formats, conjugation strategy is likely to play an increasingly decisive role in determining whether molecular complexity translates into therapeutic benefit. It should therefore be treated as an upstream translational design variable that aligns payload properties, antibody architecture, manufacturability, and CMC objectives from the outset of development.

## Figures and Tables

**Table 1:** FDA-approved antibody–drug conjugates (ADCs) as of 2 July 2026.

Approval Year	Product Name (Generic Name)	Target	Payload Class	Indications	Manufacturer	Conjugation Site	DAR	Linker Type	Ref.
2000 (FDA) withdrawn 2010; re-approved 2017	Mylotarg (Gemtuzumab ozogamicin)	CD33	DNA-cleaving agent (Calicheamicin)	AML	Pfizer (New York, NY, USA)	Lys	2–3	Hydrazone	[26]
2011	Adcetris (Brentuximab vedotin)	CD30	Microtubule inhibitor (MMAE)	HL; sALCL	Seagen (Bothell, WA, USA)/Takeda (Tokyo, Japan)	Cys	4	MC-Val-Cit PABC	[27]
2013	Kadcyla (Trastuzumab emtansine)	HER2	Microtubule inhibitor (DM1)	HER2+ breast cancer	Roche (Basel, Switzerland)	Lys	3.5	SMCC	[28]
2017	Besponsa (Inotuzumab ozogamicin)	CD22	DNA-cleaving agent (Calicheamicin)	ALL	Pfizer (New York, NY, USA)	Lys	6	Hydrazone	[29]
2019	Polivy (Polatuzumab vedotin-piiq)	CD79b	Microtubule inhibitor (MMAE)	DLBCL	Roche (Basel, Switzerland)	Cys	3.5	MC-Val-Cit PABC	[30]
2019	Padcev (Enfortumab vedotin)	Nectin-4	Microtubule inhibitor (MMAE)	mUC	Astellas (Tokyo, Japan)/Seagen (Bothell, WA, USA)	Cys	3.8	MC-Val-Cit PABC	[31]
2019	Enhertu (Trastuzumab deruxtecan)	HER2	Topoisomerase I inhibitor (Dxd)	HER2+BC; GC	AstraZeneca (Cambridge, UK)/Daiichi Sankyo (Tokyo, Japan)	Cys	8	Gly-Gly Phe-Gly	[32]
2020	Trodelvy (Sacituzumab govitecan)	Trop-2	Topoisomerase I inhibitor (SN-38)	mTNBC; mUC	Gilead (Foster City, CA, USA)	Cys	7.6	CL2A	[33]
2020 (accelerated approval); withdrawn 2023; re-approved 2025	Blenrep (Belantamab mafodotin-blmf)	BCMA	Microtubule inhibitor (MMAF)	MM	GSK (London, UK)	Cys	4	MC	[34]
2021	Zynlonta (Loncastuximab tesirine)	CD19	DNA-crosslinking agent (SG3199)	DLBCL	ADC Therapeutics (Lausanne, Switzerland)	Cys	2.3	MC-PEG8 Val-Ala PABC	[35]
2021	Tivdak (Tisotumab vedotin-tftv)	Tissue factor	Microtubule inhibitor (MMAE)	CxCa	Seagen (Bothell, WA, USA)	Cys	4	MC-Val-Cit-PABC	[36]
2022	ELAHERE (Mirvetuximab soravtansine)	FRα	Microtubule inhibitor (DM4)	OC	ImmunoGen (Waltham, MA, USA)	Lys	3.5	Sulfo-SPDB	[37]
2025	Datroway (Datopotamab Deruxtecan)	Trop-2	Topoisomerase I inhibitor (Dxd)	HR+/HER2- BC EGFR-mutated NSCLC	AstraZene-ca (Cambridge, UK)/Daiichi Sankyo (Tokyo, Japan)	Cys	4	Gly-Gly Phe-Gly	[38]
2025	EMRELIS (Telisotuzumab vedotin)	c-Met	Microtubule inhibitor (MMAE)	NSCLC	AbbVie (North Chicago, IL, USA)	Cys	4	MC-Val-Cit-PABC	[39]

Abbreviations: AML: acute myeloid leukemia; Ala: alanine; ALL: acute lymphoblastic leukemia; BC: breast cancer; Cit: citrulline; CxCa: cervical cancer; DLBCL: diffuse large B-cell lymphoma; DM1: maytansinoid DM1; DM4: maytansinoid DM4; Dxd: deruxtecan; EGFR: epidermal growth factor receptor; FRα: folate receptor alpha; GC: gastric cancer; Gly: glycine; HER2: human epidermal growth factor receptor 2; HL: Hodgkin lymphoma; HR: hormone receptor; MC: maleimidocaproyl; MM: multiple myeloma; MMAE: monomethyl auristatin E; mUC: metastatic urothelial carcinoma; mTNBC: metastatic triple-negative breast cancer; OC: ovarian cancer; PABC: *p*-aminobenzyloxycarbonyl; PEG8: polyethylene glycol 8; Phe: phenylalanine; NSCLC: non-small cell lung cancer; sALCL: systemic anaplastic large cell lymphoma; SMCC: succinimidyl 4-(*N*-maleimidomethyl) cyclohexane-1-carboxylate; Sulfo-SPDB: sulfo-*N*-succinimidyl 4-(2-pyridyldithio)butyrate; Val: valine.

**Table 2:** Selected representative ADC programs using second-generation conjugation platforms.

2nd Generation Conjugation Platform (Method)	Representative ADC	Target/Payload	Clinical Evidence/Status	Refs.
THIOMAB (engineered cysteine conjugation)	DCDS0780A	CD79b/MMAE	Published Phase I data; study completed	[41,42]
THIOMAB (engineered cysteine conjugation)	HDP-101	BCMA/Amanitin	Preliminary first-in-human data; Phase I/II recruiting	[43]
ConjuALL (CAAX/farnesyl transferase-mediated conjugation)	LCB84	Trop-2/MMAE	Phase I/II recruiting; mature outcome data remain limited	[44]
EuCODE (unnatural amino acid incorporation)	ARX788	HER2/AS269	Published early-phase data; Phase II/III development	[45,46]
XpressCF+ (cell-free unnatural amino acid incorporation)	STRO-002	FRα/SC209	Preclinical characterization; Phase I study completed	[47]
SMAC (Sortase A-mediated conjugation)	NBE-002	ROR1/PNU-159682	Preclinical evidence; Phase I/II study terminated	[48]
SMARTag (formylglycine-generating enzyme (FGE)-mediated aldehyde-tag conjugation)	TRPH-222	CD22/Maytansinoid	Preliminary Phase I tolerability and activity signal; study completed	[49]
mTG (microbial transglutaminase-mediated conjugation)	DP303c	HER2/MMAE	Preclinical and preliminary first-in-human evidence; Phase I/II development	[45,50]

Abbreviations: BCMA: B-cell maturation antigen; FRα: folate receptor alpha; HER2: human epidermal growth factor receptor 2; MMAE: monomethyl auristatin E; ROR1: receptor tyrosine kinase-like orphan receptor 1; Trop-2: tumor-associated calcium signal transducer 2. Note: Selected examples are shown. Complete program-level information, including indications, sponsors, trial identifiers, and registry status, is provided in Supplementary Table S1. Clinical phase and registry status were verified using ClinicalTrials.gov as of 2 July 2026. Registry status refers to the listed study and does not necessarily indicate continuation or discontinuation of the overall development program.

**Table 3:** Selected representative ADC programs using third-generation conjugation strategies.

Conjugation Platform (Method)	Representative ADC	Target/Payload	Clinical Evidence/Status	Refs.
GlycoConnect (Fc glycan remodeling)	IBI343	CLDN18.2/Exatecan	Published Phase I data; Phase III recruiting	[82,83,84]
GlycoConnect (Fc glycan remodeling)	MRG004A	Tissue factor/MMAE	Preliminary Phase I/II findings; study completed	[85,86]
AbClick (proximity-induced Lys248 conjugation)	AT-211 (DA-3501)	CLDN18.2/MMAE	First-in-human Phase I/II recruiting; mature clinical outcomes data remain limited	[87]
ThioBridge (disulfide rebridging)	MBRC-101	EphA5/MMAE	Phase I/II recruiting; limited public clinical outcome data	[88]
IDconnect (disulfide rebridging)	9MW2821	Nectin-4/MMAE	Published early-phase evidence; Phase III recruiting	[89]

Abbreviations: CLDN: claudin; EphA: ephrin type-A receptor; MMAE: monomethyl auristatin E. Note: Selected examples are shown. Complete program-level information, including indications, sponsors, trial identifiers, and registry status, is provided in Supplementary Table S2. Clinical phase and registry status were verified using ClinicalTrials.gov as of 2 July 2026. Registry status refers to the listed study and does not necessarily indicate continuation or discontinuation of the overall development program.

**Table 4:** Practical selection considerations for ADC conjugation strategies.

Conjugation Strategy	Representative Technologies	Typical DAR	Product Homogeneity	Antibody Engineering Required	Clinical Maturity	Practical Strengths	Key Constraints
Lysine-based stochastic conjugation	NHS ester chemistry	Broad, variable distribution	Low	No	Multiple FDA-approved ADCs	Simple, scalable, native compatible	High heterogeneity; antigen-binding risk; batch variability
Cysteine-based stochastic conjugation	Interchain disulfide reduction + maleimide coupling	2, 4, 6, 8	Moderate	No	Multiple FDA-approved ADCs	Clinically validated with moderate DAR control	Disulfide disruption; maleimide instability; retro-Michael deconjugation
Engineered cysteine conjugation	THIOMAB	2	High	Yes	Clinical-stage ADCs	Defined sites with tighter DAR control	Engineering burden; disulfide scrambling risk
Unnatural amino acid incorporation	EuCODE, XpressCF+	2–4	Very high	Yes	Clinical-stage ADCs	Precise bioorthogonal payload placement	Yield challenges; immunogenicity risk; specialized systems
Enzymatic tagging	SMARTag, SMAC, mTG, ConjuALL	2–4	Very high	Yes	Clinical-stage ADCs	High selectivity under mild conditions	Engineered motifs required; enzyme efficiency constraints
Glycan remodeling	GlycoConnect	2–4	High	No	Clinical-stage ADCs	Improved homogeneity with native sequence retention	Enzymatic processing complexity; glycan management
Proximity-induced lysine modification	AJICAP, AbClick, Z-M platform	2	Very high	No	Early clinical-stage ADCs	Controlled, traceless native antibody conjugation	Reagent complexity; scalability/accessibility issues
Disulfide rebridging	ThioBridge, IDconnect, WuXiDAR4	4	High	No	Clinical-stage ADCs	Improved stability and homogeneity with native antibody compatibility	Controlled reduction required; linker chemistry limitations

Abbreviations: ADCs: antibody–drug conjugates; DARs: drug-to-antibody ratios; FDA: Food and Drug Administration; NHS: *N*-hydroxysuccinimide.

**Table 5:** Key regulatory expectations and CMC relevance of ADC quality attributes.

Attribute	Description	Regulatory Impact	CMC/Analytical Relevance
DAR heterogeneity	Variability in payload per antibody beyond average DARs	Affects batch consistency, dosing precision, and safety	Complicates DAR profiling, site occupancy analysis, and comparability assessment
Aggregation	Self-association of ADCs after conjugation	Elevates immunogenicity risk, reduces bioavailability	Requires aggregate monitoring, stability control, and release testing
Off-target toxicity	Premature or nonspecific release of payload	Narrow therapeutic index	Linked to linker stability, deconjugation risk, and structurally stressed species
Manufacturability	Reproducibility and robustness of conjugation chemistry	Influences scalability and validation requirements	Affects process robustness, lot-to-lot consistency, and validation strategy

Abbreviations: ADCs: antibody–drug conjugates; CMC: chemistry, manufacturing, and controls; DARs: drug-to-antibody ratios.

## Data Availability

No new data were created or analyzed in this study. Data sharing is not applicable to this article.

## Supplementary Materials

The following supporting information can be downloaded at: https://www.mdpi.com/article/10.3390/pharmaceutics18070852/s1, Table S1: Complete program-level listing of ADCs using second-generation conjugation platforms; Table S2: Complete program-level listing of ADCs using third-generation conjugation strategies. References [6,41,42,43,44,45,46,47,48,49,50,54,55,82,83,84,85,86,87,88,89,122,123,124,125,126,127,128,129,130,131,132,133,134,135,136,137,138,139,140,141,142] cited in Supplementary Materials.

## Abbreviations

The following abbreviations are used in this manuscript:

ADCs	Antibody–drug conjugates
AF-HPA	Auristatin F hydroxypropylamide
Ala	Alanine
ALL	Acute lymphoblastic leukemia
AML	Acute myeloid leukemia
AzK	*N*ε-azido-lysine
AST	Advanced solid tumor
BC	Breast cancer
BCMs	B-cell malignancies
BCMA	B-cell maturation antigen
BCL	B-cell lymphoma
B-NHL	B-cell non-Hodgkin lymphoma
CLDN	Claudin
cGMP	Current good manufacturing practice
cGU	Cancer of genitourinary
Cit	Citrulline
CMC	Chemistry, manufacturing, and controls
CHO cells	Chinese hamster ovary cells
CRPC	Castration-resistant prostate cancer
CxCa	Cervical cancer
cUADT	Cancer of upper aerodi-gestive tract
DARs	Drug-to-antibody ratios
DLBCL	Diffuse large B-cell lymphoma
DM	Maytansinoid DM
Dxd	Deruxtecan
EC	Endometrial cancer
EGFR	Epidermal growth factor receptor
EOC	Epithelial ovarian cancer
EphA	Ephrin type-A receptor
ESC	Esophageal cancer
FcRn	Neonatal Fc receptor
FDA	Food and Drug Administration
FGE	Formylglycine-generating enzyme
FRα	Folate receptor alpha
GAC	Gastric adenocarcinoma
GC	Gastric cancer
GEJAC	Gastroesophageal junction adenocarcinoma
Gly	Glycine
HER2	Human epidermal growth factor receptor 2
HIC-HPLC	Hydrophobic interaction chromatography–high-performance liquid chromatography
HL	Hodgkin lymphoma
HR	Hormone receptor
LA/mAST	Locally advanced or metastatic solid tumors
LC	Lung cancer
LC–MS	Liquid chromatography–mass spectrometry
mAbs	Monoclonal antibodies
mBC	Metastatic breast cancer
MC	Maleimidocaproyl
mCC	Metastatic cervical cancer
mCRPC	Metastatic castration-resistant prostate cancer
MDS	Myelodysplastic syndrome
mGC	Metastatic gastric cancer
MM	Multiple myeloma
MMAE	Monomethyl auristatin E
mTG	Microbial transglutaminase
mTNBC	Metastatic triple-negative breast cancer
mUC	Metastatic urothelial carcinoma
NHS	*N*-hydroxysuccinimide
NSCLC	Non-small cell lung cancer
OC	Ovarian cancer
PABC	*p*-aminobenzyloxycarbonyl
pAcF	*P*-acetylphenylalanine
PC	Pancreatic cancer
PCD	Plasma cell disorders
PDAC	Pancreatic ductal adenocarcinoma
PEG	Polyethylene glycol
Phe	Phenylalanine
PROC	Platinum-resistant ovarian cancer
ROR1	Receptor tyrosine kinase-like orphan receptor 1
R/R	Relapsed/refractory
sALCL	Systemic anaplastic large cell lymphoma
SCLC	Small cell lung cancer
SEC	Size-exclusion chromatography
SMCC	Succinimidyl 4-(*N*-maleimidomethyl) cyclohexane-1-carboxylate
SPAAC	Strain-promoted azide–alkyne cycloaddition
Sulfo-SPDB	Sulfo-*N*-succinimidyl 4-(2-pyridyldithio)butyrate
T-DXd	Trastuzumab deruxtecan
TNBC	Triple-negative breast cancer
Trop-2	Tumor-associated calcium signal transducer 2
UAA	Unnatural amino acid
UPC	Unresectable pancreatic cancer
Val	Valine

## References

[1] Beck A., Goetsch L., Dumontet C., Corvaïa N. (2017). Strategies and challenges for the next generation of antibody–drug conjugates. Nat. Rev. Drug Discov..

[2] Sievers E.L., Senter P.D. (2013). Antibody–drug conjugates in cancer therapy. Annu. Rev. Med..

[3] Modi S., Jacot W., Yamashita T., Sohn J., Vidal M., Tokunaga E., Tsurutani J., Ueno N.T., Prat A., Chae Y.S. (2022). Trastuzumab deruxtecan in previously treated HER2-low advanced breast cancer. N. Engl. J. Med..

[4] Junutula J.R., Raab H., Clark S., Bhakta S., Leipold D.D., Weir S., Chen Y., Simpson M., Tsai S.P., Dennis M.S. (2008). Site-specific conjugation of a cytotoxic drug to an antibody improves therapeutic index. Nat. Biotechnol..

[5] Strop P., Liu S.H., Dorywalska M., Delaria K., Dushin R.G., Tran T.T., Ho W.H., Farias S., Casas M.G., Abdiche Y. (2013). Location matters: Site of conjugation modulates stability and pharmacokinetics of antibody drug conjugates. Chem. Biol..

[6] Fan Q., Chen H., Wei G., Wei D., Wang Z., Zhang L., Wang J., Zhu M. (2025). A review of conjugation technologies for antibody–drug conjugates. Antib. Ther..

[7] Walsh S.J., Bargh J.D., Dannheim F.M., Hanby A.R., Seki H., Counsell A.J., Ou X., Fowler E., Ashman N., Takada Y. (2021). Site-selective modification strategies in antibody–drug conjugates. Chem. Soc. Rev..

[8] Shen B.Q., Xu K., Liu L., Raab H., Bhakta S., Kenrick M., Reponte K.L.P., Tien J., Yu S.F., Mai E. (2012). Conjugation site modulates the in vivo stability and therapeutic activity of antibody–drug conjugates. Nat. Biotechnol..

[9] U.S. Food and Drug Administration (2024). Clinical Pharmacology Considerations for Antibody-Drug Conjugates: Guidance for Industry. https://www.fda.gov/regulatory-information/search-fda-guidance-documents/clinical-pharmacology-considerations-antibody-drug-conjugates-guidance-industry.

[10] Brun M.P., Gauzy-Lazo L. (2013). Protocols for lysine conjugation. Methods Mol. Biol..

[11] Koniev O., Wagner A. (2015). Developments and recent advancements in the field of endogenous amino acid selective bond forming reactions for bioconjugation. Chem. Soc. Rev..

[12] Arlotta K.J., Gandhi A.V., Chen H.-N., Nervig C.S., Carpenter J.F., Owen S.C. (2018). In-depth comparison of lysine-based antibody-drug conjugates prepared on solid support versus in solution. Antibodies.

[13] Hamann P.R., Hinman L.M., Hollander I., Beyer C.F., Lindh D., Holcomb R., Hallett W., Tsou H.R., Upeslacis J., Shochat D. (2002). Gemtuzumab ozogamicin, a potent and selective anti-CD33 antibody–calicheamicin conjugate for treatment of acute myeloid leukemia. Bioconjugate Chem..

[14] Lambert J.M., Chari R.V.J. (2014). Ado-trastuzumab emtansine (T-DM1): An antibody–drug conjugate (ADC) for HER2-positive breast cancer. J. Med. Chem..

[15] DiJoseph J.F., Armellino D.C., Boghaert E.R., Khandke K., Dougher M.M., Sridharan L., Kunz A., Hamann P.R., Gorovits B., Udata C. (2004). Antibody-targeted chemotherapy with CMC-544: A CD22-targeted immunoconjugate of calicheamicin for the treatment of B-lymphoid malignancies. Blood.

[16] Fu Z., Li S., Han S., Shi C., Zhang Y. (2022). Antibody–drug conjugate: The “biological missile” for targeted cancer therapy. Signal Transduct. Target. Ther..

[17] Moore E.J., Rice M., Roy G., Zhang W., Marelli M. (2024). Emerging conjugation strategies and protein engineering technologies aim to improve ADCs in the fight against cancer. Xenobiotica.

[18] Ohri R., Bhakta S., Fourie-O’Donohue A., Dela Cruz-Chuh J., Tsai S.P., Cook R., Wei B., Ng C., Wong A.W., Bos A.B. (2018). High-throughput cysteine scanning to identify stable antibody conjugation sites for maleimide- and disulfide-based linkers. Bioconjugate Chem..

[19] Lyon R.P., Bovee T.D., Doronina S.O., Burke P.J., Hunter J.H., Neff-Laford H.D., Jonas M., Anderson M.E., Setter J.R., Senter P.D. (2015). Reducing hydrophobicity of homogeneous antibody–drug conjugates improves pharmacokinetics and therapeutic index. Nat. Biotechnol..

[20] Wang R., Hu B., Pan Z., Mo C., Zhao X., Liu G., Hou P., Cui Q., Xu Z., Wang W. (2025). Antibody–drug conjugates (ADCs): Current and future biopharmaceuticals. J. Hematol. Oncol..

[21] Liu K., Li M., Li Y., Li Y., Chen Z., Tang Y., Yang M., Deng G., Liu H. (2024). A review of the clinical efficacy of FDA-approved antibody–drug conjugates in human cancers. Mol. Cancer.

[22] Gogia P., Ashraf H., Bhasin S., Xu Y. (2023). Antibody–drug conjugates: A review of approved drugs and their clinical level of evidence. Cancers.

[23] U.S. Food and Drug Administration Novel Drug Approvals for 2025. https://www.fda.gov/drugs/novel-drug-approvals-fda/novel-drug-approvals-2025.

[24] Lu D., Zhang D., Jin J.Y., Li C., Chen Y. (2026). Characterization of drug–drug interaction for antibody–drug conjugates and risk assessment using various approaches including physiologically based pharmacokinetic modeling. Drug Metab. Pharmacokinet..

[25] Alradwan I.A., Alnefaie M.K., Al Fayez N., Aodah A.H., Majrashi M.A., Alturki M., Fallatah M.M., Almughem F.A., Tawfik E.A., Alshehri A.A. (2025). Strategic and chemical advances in antibody–drug conjugates. Pharmaceutics.

[26] Norsworthy K.J., Ko C.W., Lee J.E., Liu J., John C.S., Przepiorka D., Farrell A.T., Pazdur R. (2018). FDA Approval Summary: Mylotarg for Treatment of Patients with Relapsed or Refractory CD33-Positive Acute Myeloid Leukemia. Oncologist.

[27] Richardson N.C., Kasamon Y.L., Chen H., de Claro R.A., Ye J., Blumenthal G.M., Farrell A.T., Pazdur R. (2019). FDA Approval Summary: Brentuximab Vedotin in First-Line Treatment of Peripheral T-Cell Lymphoma. Oncologist.

[28] Wedam S., Fashoyin-Aje L., Gao X., Bloomquist E., Tang S., Sridhara R., Goldberg K.B., King-Kallimanis B.L., Theoret M.R., Ibrahim A. (2020). FDA Approval Summary: Ado-Trastuzumab Emtansine for the Adjuvant Treatment of HER2-positive Early Breast Cancer. Clin. Cancer Res..

[29] Tvito A., Rowe J.M. (2017). Inotuzumab ozogamicin for the treatment of acute lymphoblastic leukemia. Expert. Opin. Biol. Ther..

[30] Deeks E.D. (2019). Polatuzumab Vedotin: First Global Approval. Drugs.

[31] Chang E., Weinstock C., Zhang L., Charlab R., Dorff S.E., Gong Y., Hsu V., Li F., Ricks T.K., Song P. (2021). FDA Approval Summary: Enfortumab Vedotin for Locally Advanced or Metastatic Urothelial Carcinoma. Clin. Cancer Res..

[32] Modi S. (2021). Trastuzumab Deruxtecan in Previously Treated HER2-Positive Metastatic Breast Cancer: Plain Language Summary of the DESTINY-Breast01 Study. Future Oncol..

[33] Spring L.M., Nakajima E., Hutchinson J., Viscosi E., Blouin G., Weekes C., Rugo H., Moy B., Bardia A. (2021). Sacituzumab Govitecan for Metastatic Triple-Negative Breast Cancer: Clinical Overview and Management of Potential Toxicities. Oncologist.

[34] Ketchum E.B., Clarke A., Clemmons A.B. (2022). Belantamab Mafodotin-blmf: A Novel Antibody-Drug Conjugate for Treatment of Patients with Relapsed/Refractory Multiple Myeloma. J. Adv. Pract. Oncol..

[35] Furqan F., Hamadani M. (2022). Loncastuximab tesirine in relapsed or refractory diffuse large B-cell lymphoma: A review of clinical data. Ther. Adv. Hematol..

[36] De S.K. (2022). Tisotumab Vedotin: The First FDA-Approved Antibody-Drug Conjugate for Cervical Cancer. Anticancer Agents Med. Chem..

[37] Dilawari A., Shah M., Ison G., Gittleman H., Fiero M.H., Shah A., Hamed S.S., Qiu J., Yu J., Manheng W. (2023). FDA Approval Summary: Mirvetuximab Soravtansine-Gynx for FRα-Positive, Platinum-Resistant Ovarian Cancer. Clin. Cancer Res..

[38] Royce M., Shah M., Zhang L., Cheng J., Bonner M.K., Pegues M., Miller C.P., Leu L., Price L.S.L., Qiu J. (2025). FDA Approval Summary: Datopotamab Deruxtecan-dlnk for Treatment of Patients with Unresectable or Metastatic, HR-Positive, HER2-Negative Breast Cancer. Clin. Cancer Res..

[39] Zhao C., Lu D., Gao J. (2025). Telisotuzumab vedotin: The first-in-class c-Met-targeted antibody-drug conjugate granted FDA accelerated approval for treatment of non-squamous non-small cell lung cancer (NSCLC). Drug Discov. Ther..

[40] Junutula J.R., Flagella K.M., Graham R.A., Parsons K.L., Ha E., Raab H., Bhakta S., Nguyen T., Dugger D.L., Li G. (2010). Engineered thio-trastuzumab-DM1 conjugate with an improved therapeutic index to target human epidermal growth factor receptor 2–positive breast cancer. Clin. Cancer Res..

[41] ClinicalTrials.gov A Study of Escalating Doses of DCDS0780A in Participants with B-Cell Non-Hodgkin Lymphoma. Identifier: NCT02453087. NCT02453087.

[42] Herrera A.F., Patel M.R., Burke J.M., Advani R., Cheson B.D., Sharman J.P., Penuel E., Polson A.G., Liao C.D., Li C. (2022). Anti-CD79B antibody–drug conjugate DCDS0780A in patients with B-cell non-Hodgkin lymphoma: Phase 1 dose-escalation study. Clin. Cancer Res..

[43] Orlowski R.Z., Richard S., Kaufman J.L., Grosicki S., Takacs I., Strassz A., Pahl A.M., Kulke M., Michael T., Last A. (2024). The anti-BCMA antibody–drug conjugate HDP-101 with a novel amanitin payload shows promising initial first-in-human results in relapsed multiple myeloma. Blood.

[44] Liu X., Ma L., Li J., Sun L., Yang Y., Liu T., Xing D., Yan S., Zhang M. (2024). Trop2-targeted therapies in solid tumors: Advances and future directions. Theranostics.

[45] Najminejad Z., Dehghani F., Mirzaei Y., Mer A.H., Saghi S.A., Abdolvahab M.H., Bagheri N., Meyfour A., Jafari A., Jahandideh S. (2023). Clinical perspective: Antibody–drug conjugates for the treatment of HER2-positive breast cancer. Mol. Ther..

[46] Niu N., Xue J., Chen G., Qiu F., Xu Q., Zheng X., Liu C., Zhao Y., Gu X., Zhao Y. (2025). Neoadjuvant ARX788 plus pyrotinib versus trastuzumab, pertuzumab, docetaxel and carboplatin for HER2-positive breast cancer: A randomized phase 2b trial. Nat. Commun..

[47] Li X., Zhou S., Abrahams C.L., Krimm S., Smith J., Bajjuri K., Stephenson H.T., Henningsen R., Hanson J., Heibeck T.H. (2023). Discovery of STRO-002, a novel homogeneous ADC targeting folate receptor alpha for the treatment of ovarian and endometrial cancers. Mol. Cancer Ther..

[48] ClinicalTrials.gov NBE-002 in Patients with Advanced Solid Tumors. Identifier: NCT04441099. NCT04441099.

[49] Hernandez-Ilizaliturri F.J., Flinn I.W., Kuruvilla J., Assouline S.E., Ulrickson M.L., Christian B.A., Landsburg D.J., Stuart M., Lowman H., Levin N. (2020). A phase I pharmacokinetic (PK) and safety study of Trph-222 in patients with relapsed/refractory B-cell non-Hodgkin lymphoma (R/R NHL): Dose-escalation results. Blood.

[50] Zhang J., Du Y., Meng Y., Liu X., Mu Y., Liu Y., Shi Y., Wang J., Zang A., Gu S. (2024). First-in-human study of DP303c, a HER2-targeted antibody-drug conjugate in patients with HER2 positive solid tumors. npj Precis. Oncol..

[51] Adhikari P., Zacharias N., Ohri R., Sadowsky J., Tumey L.N. (2020). Site-specific conjugation to Cys-engineered THIOMAB™ antibodies. Antibody-Drug Conjugates: Methods and Protocols.

[52] Kung Sutherland M.S., Walter R.B., Jeffrey S.C., Burke P.J., Yu C., Kostner H., Stone I., Ryan M.C., Sussman D., Lyon R.P. (2013). SGN-CD33A: A novel CD33-targeting antibody–drug conjugate using a pyrrolobenzodiazepine dimer is active in models of drug-resistant AML. Blood.

[53] Kaempffe A., Dickgiesser S., Rasche N., Paoletti A., Bertotti E., De Salve I., Federico R.S., Sirtori F.R., Kellner R., Könning D. (2021). Effect of conjugation site and technique on the stability and pharmacokinetics of antibody–drug conjugates. J. Pharm. Sci..

[54] Dimopoulos M.A., Migkou M., Bhutani M., Ailawadhi S., Kalff A., Walcott F.L., Pore N., Brown M., Wang F., Cheng L.I. (2024). Phase 1 first-in-human study of MEDI2228, a BCMA-targeted ADC, in patients with relapsed refractory multiple myeloma. Leuk. Lymphoma.

[55] Kotecki N., van Herpen C.M.L., Curigliano C., Hendriks M., Vermaas T.C., Corrigan L., Belli C., Jungels C., Desar I.M.E., Koper N.P. (2023). Abstract CT185: First-in-human dose-escalation trial with the c-MET-targeting antibody–drug conjugate BYON3521. Cancer Res..

[56] Zimmerman E.S., Heibeck T.H., Gill A., Li X., Murray C.J., Madlansacay M.R., Tran C., Uter N.T., Yin G., Rivers P.J. (2014). Production of site-specific antibody–drug conjugates using optimized non-natural amino acids in a cell-free expression system. Bioconjugate Chem..

[57] Maloney R., Buuh Z.Y., Zhao Y., Wang R.E. (2020). Site-specific antibody fragment conjugates for targeted imaging. Methods Enzymol..

[58] Hanson J., Groff D., Carlos A., Usman H., Fong K., Yu A., Armstrong S., Dwyer A., Masikat M.R., Yuan D. (2023). An integrated in vivo/in vitro protein production platform for site-specific antibody drug conjugates. Bioengineering.

[59] Zhou Q. (2017). Site-specific antibody conjugation for ADC and beyond. Biomedicines.

[60] Axup J.Y., Bajjuri K.M., Ritland M., Hutchins B.M., Kim C.H., Kazane S.A., Halder R., Forsyth J.S., Santidrian A.F., Stafin K. (2012). Synthesis of site-specific antibody–drug conjugates using unnatural amino acids. Proc. Natl. Acad. Sci. USA.

[61] Tian F., Lu Y., Manibusan A., Sellers A., Tran H., Sun Y., Phuong T., Barnett R., Hehli B., Song F. (2014). A general approach to site-specific antibody drug conjugates. Proc. Natl. Acad. Sci. USA.

[62] Jackson D., Atkinson J., Guevara C.I., Zhang C., Kery V., Moon S.J., Virata C., Yang P., Lowe C., Pinkstaff J. (2014). In vitro and in vivo evaluation of cysteine and site-specific conjugated Herceptin antibody–drug conjugates. PLoS ONE.

[63] Skidmore L., Sakamuri S., Knudsen N.A., Hewet A.G., Milutinovic S., Barkho W., Biroc S.L., Kirtley J., Marsden R., Kristine S. (2020). ARX788, a site-specific anti-HER2 antibody–drug conjugate, demonstrates potent and selective activity in HER2-low and T-DM1–resistant breast and gastric cancers. Mol. Cancer Ther..

[64] Hu X., Wang L., Zhang J., Zhang Q., Ouyang Q., Wang X., Li W., Xie W., Tong Z., Xu F. (2024). ACE-Breast-02: A pivotal phase II/III trial of ARX788, a novel anti-HER2 antibody–drug conjugate (ADC), versus lapatinib plus capecitabine for HER2-positive advanced breast cancer (ABC). J. Clin. Oncol..

[65] Zhang J., Ji D., Shen W., Xiao Q., Gu Y., O’Shaughnessy J., Hu X. (2022). Phase I trial of a novel anti-HER2 antibody–drug conjugate, ARX788, for the treatment of HER2-positive metastatic breast cancer. Clin. Cancer Res..

[66] Díaz-Rodríguez E., Gandullo-Sánchez L., Ocaña A., Pandiella A. (2022). Novel ADCs and strategies to overcome resistance to anti-HER2 ADCs. Cancers.

[67] Yin Q., Zhang Y., Xie X., Hou M., Chen X., Ding J. (2025). Navigating the future of gastric cancer treatment: A review on the impact of antibody–drug conjugates. Cell Death Discov..

[68] Jun J.S., Hong S., Park J.H., Shin J., Lee D.H. (2025). Automated and programmable cell-free systems for scalable synthetic biology with a focus on biofoundry integration. J. Microbiol. Biotechnol..

[69] Falck G., Müller K.M. (2018). Enzyme-based labeling strategies for antibody–drug conjugates and antibody mimetics. Antibodies.

[70] Adumeau P., Sharma S.K., Brent C., Zeglis B.M. (2016). Site-specifically labeled immunoconjugates for molecular imaging—Part 2: Peptide tags and unnatural amino acids. Mol. Imaging Biol..

[71] Popp M.W., Antos J.M., Grotenbreg G.M., Spooner E., Ploegh H.L. (2007). Sortagging: A versatile method for protein labeling. Nat. Chem. Biol..

[72] Beerli R.R., Hell T., Merkel A.S., Grawunder U. (2015). Sortase enzyme-mediated generation of site-specifically conjugated antibody drug conjugates with high in vitro and in vivo potency. PLoS ONE.

[73] Liu D., Vandenberg C.J., Sini P., Waldmeier L., Baumgartinger R., Pisarsky L., Petroczi G., Ratnayake G., Scott C.L., Ford C.E. (2025). The antibody–drug conjugate targeting ROR1, NBE-002, is active in high-grade serous ovarian cancer preclinical models. Ther. Adv. Med. Oncol..

[74] Dierks T., Dickmanns A., Preusser-Kunze A., Schmidt B., Mariappan M., von Figura K., Ficner R., Rudolph M.G. (2005). Molecular basis for multiple sulfatase deficiency and mechanism for formylglycine generation of the human formylglycine-generating enzyme. Cell.

[75] Carrico I.S., Carlson B.L., Bertozzi C.R. (2007). Introducing genetically encoded aldehydes into proteins. Nat. Chem. Biol..

[76] Drake P.M., Albers A.E., Baker J., Banas S., Barfield R.M., Bhat A.S., de Hart G.W., Garofalo A.W., Holder P., Jones L.C. (2014). Aldehyde tag coupled with HIPS chemistry enables the production of ADCs conjugated site-specifically to different antibody regions with distinct in vivo efficacy and PK outcomes. Bioconjugate Chem..

[77] Dickgiesser S., Deweid L., Kellner R., Kolmar H., Rasche N. (2019). Site-specific antibody–drug conjugation using microbial transglutaminase. Methods Mol. Biol..

[78] Schumacher D., Hackenberger C.P.R., Leonhardt H., Helma J. (2016). Current status: Site-specific antibody drug conjugates. J. Clin. Immunol..

[79] Jeger S., Zimmermann K., Blanc A., Grünberg J., Honer M., Hunziker P., Struthers H., Schibli R. (2010). Site-specific and stoichiometric modification of antibodies by bacterial transglutaminase. Angew. Chem. Int. Ed..

[80] Beitello E., Osei K., Breausche F.E., Friesen J.A., Driskell J.D. (2026). Scope of microbial transglutaminase for site-specific and oriented immobilization of native antibodies from various host species. Langmuir.

[81] Hui X., Yuan C., Cao W., Ge W., Zhang D., Dan M., Zhao Q., Liu B., Liu B. (2022). An innovative site-specific anti-HER2 antibody–drug conjugate with high homogeneity and improved therapeutic index. OncoTargets Targets Ther..

[82] Liu J., Yang J., Sun Y., Gong J., Yue J., Pan Y., Sun M., Song R., Xiao X., Tazbirkova A. (2025). CLDN18.2-targeting antibody–drug conjugate IBI343 in advanced gastric or gastroesophageal junction adenocarcinoma: A phase 1 trial. Nat. Med..

[83] Yu X., Zhang J., Tazbirkova A., Yang J., Yue J., Sun Y., Pan Y., Sun M., Qin Y., Shen L. (2024). Safety and efficacy of IBI343 (anti-claudin18.2 antibody–drug conjugate) in patients with advanced pancreatic ductal adenocarcinoma or biliary tract cancer: Preliminary results from a phase 1 study. J. Clin. Oncol..

[84] Yu X., Zhang J., Liu J.J., Yang J., Yue J., Sun Y., Pan Y., Sun M.L., Qin Y., Shen L. (2024). 132MO Anti-claudin18.2 (CLDN18.2) antibody–drug conjugate (ADC) IBI343 in patients with advanced pancreatic ductal adenocarcinoma (PDAC): Updated results from a phase I study. Ann. Oncol..

[85] Park W., Zhang J., Dayyani F., Shan J., Liu R., Guo R., O’Reilly E.M., Liu Z., Gao S., Wu X. (2024). Phase I/II first-in-human study to evaluate the safety and efficacy of tissue factor ADC MRG004A in patients with solid tumors. J. Clin. Oncol..

[86] Ren W., Park W., Yu X., Shan J., Wu H., Zhao H., Hu C., Tang J., Yang J., Niu Z. (2025). Tissue factor (TF) antibody–drug conjugate (ADC) MRG004A in patients (pts) with advanced pancreatic cancer (PC): Updated results from a phase I study. Ann. Oncol..

[87] ClinicalTrials.gov A First-In-Human Phase I/IIa Study to Evaluate DA 3501 in Patients with Advanced Gastric or Gastro-Esophageal Junction Adenocarcinoma and Pancreatic Ductal Adenocarcinoma. Identifier: NCT07481357. NCT07481357.

[88] Sen S., Salkeni M.A., Albany C., Gandhi N.J., Edenfield W.J., Parsons K., Chen I., Powderly J.D. (2024). A multi-center, open-label phase 1/1b dose finding, safety, and pharmacokinetic study of MBRC-101, an anti-EphA5 monomethyl auristatin (MMAE) antibody drug conjugate, in advanced refractory solid tumors. J. Clin. Oncol..

[89] Zhang J., Liu R., Wang S., Feng Z., Yang H., Gao S., Li X., Yao X., Chen J., Gong Z. (2025). Bulumtatug fuvedotin (BFv, 9MW2821), a next-generation nectin-4–targeting antibody–drug conjugate, in patients with advanced solid tumors: A first-in-human, open-label, multicenter phase I/II study. Ann. Oncol..

[90] Van Geel R., Wijdeven M.A., Heesbeen R., Verkade J.M.M., Wasiel A.A., van Berkel S.S., van Delft F.L. (2015). Chemoenzymatic conjugation of toxic payloads to the globally conserved N-glycan of native mAbs provides homogeneous and highly efficacious antibody–drug conjugates. Bioconjugate Chem..

[91] Wijdeven M.A., van Geel R., Hoogenboom J.H., Verkade J.M.M., Janssen B.M.G., Hurkmans I., de Bever L., van Berkel S.S., van Delft F.L. (2022). Enzymatic glycan remodeling–metal-free click (GlycoConnect™) provides homogeneous antibody–drug conjugates with improved stability and therapeutic index without sequence engineering. mAbs.

[92] Verkade J.M.M., Wijdeven M.A., van Geel R., Janssen B.M.G., van Berkel S.S., van Delft F.L. (2018). A polar sulfamide spacer significantly enhances the manufacturability, stability, and therapeutic index of antibody–drug conjugates. Antibodies.

[93] Zammarchi F., Havenith K.E.G., Chivers S., Hogg P., Bertelli F., Tyrer P., Janghra N., Reinert H.W., Hartley J.A., van Berkel P.H. (2022). Preclinical development of ADCT-601, a novel pyrrolobenzodiazepine dimer-based antibody–drug conjugate targeting AXL-expressing cancers. Mol. Cancer Ther..

[94] Zhou S., Yao X., Guan J., Fei K., Lu J., Wu W., Liu Y., Zhu T., Liao Z., Chen S. (2024). Abstract LB057: Pre-clinical characterization of IBI343, a site-specifically conjugated anti-claudin18.2 ADC, for treating solid tumors. Cancer Res..

[95] de Bever L., Popal S., van Schaik J., Rubahamya B., van Delft F.L., Thurber G.M., van Berkel S.S. (2023). Generation of DAR1 antibody–drug conjugates for ultrapotent payloads using tailored GlycoConnect technology. Bioconjugate Chem..

[96] Maecker H., Jonnalagadda V., Bhakta S., Jammalamadaka V., Junutula J.R. (2023). Exploration of the antibody-drug conjugate clinical landscape. mAbs.

[97] Fujii T., Matsuda Y., Seki T., Shikida N., Iwai Y., Ooba Y., Takahashi K., Isokawa M., Kawaguchi S., Hatada N. (2023). AJICAP second generation: Improved chemical site-specific conjugation technology for antibody–drug conjugate production. Bioconjugate Chem..

[98] Watanabe T., Fujii T., Stofleth J.T., Takasugi R., Takahashi K., Matsuda Y. (2023). Scale-up synthesis of site-specific antibody–drug conjugates using AJICAP second-generation technology. Org. Process Res. Dev..

[99] Lee T., Kim J.H., Kwon S.J., Seo J.W., Park S.H., Kim J., Jin J., Hong J.H., Kang H.J., Sharma C. (2022). Site-selective antibody–drug conjugation by a proximity-driven S to N acyl transfer reaction on a therapeutic antibody. J. Med. Chem..

[100] Kwon S.J., Son J., Chung S.J. (2025). Site-selective anti–PD-L1 antibody–MMAE conjugate for enhanced NSCLC therapy. ACS Med. Chem. Lett..

[101] Kim S., Kim S., Kim S., Kim N., Lee S.W., Yi H., Lee S., Sim T., Kwon Y., Lee H.S. (2024). Affinity-directed site-specific protein labeling and its application to antibody–drug conjugates. Adv. Sci..

[102] You J., Zhang J., Wang J., Jin M. (2021). Cysteine-based coupling: Challenges and solutions. Bioconjugate Chem..

[103] Ji A., Sun C., He W. (2020). Process for Preparing Antibody–Drug Conjugates with Improved Homogeneity.

[104] Badescu G., Bryant P., Bird M., Henseleit K., Swierkosz J., Parekh V., Tommasi R., Pawlisz E., Jurlewicz K., Farys M. (2014). Bridging disulfides for stable and defined antibody drug conjugates. Bioconjugate Chem..

[105] Bryant P., Pabst M., Badescu G., Bird M., McDowell W., Jamieson E., Swierkosz J., Jurlewicz K., Tommasi R., Henseleit K. (2015). In vitro and in vivo evaluation of cysteine rebridged trastuzumab–MMAE antibody–drug conjugates with defined drug-to-antibody ratios. Mol. Pharm..

[106] Pabst M., McDowell W., Manin A., Kyle A., Camper N., De Juan E., Parekh V., Rudge F., Makwana H., Kantner T. (2017). Modulation of drug–linker design to enhance in vivo potency of homogeneous antibody–drug conjugates. J. Control. Release.

[107] Staquicini F.I., Tang F.H.F., Oliveira V., Kim S.Y., Chen E.R., Markosian C., Staquicini D.I., Wu Y., Parsons J.K., Barnhart K.F. (2025). First-generation and preclinical evaluation of an EphA5-targeted antibody–drug conjugate in solid tumors. J. Clin. Investig..

[108] Zhou W., Zhu H., Wang Z., Xu H., Tan X. (2021). Preparation Method for Bis-Substituted Bridging Antibody–Drug Conjugate.

[109] Fang P., You M., Cao Y., Feng Q., Shi L., Wang J., Sun X., Yu D., Zhou W., Yin L. (2024). Development and validation of bioanalytical assays for the quantification of 9MW2821, a nectin-4–targeting antibody–drug conjugate. J. Pharm. Biomed. Anal..

[110] Zhou W., Fang P., Yu D., Ren H., You M., Yin L., Mei F., Zhu H., Wang Z., Xu H. (2023). Preclinical evaluation of 9MW2821, a site-specific monomethyl auristatin E–based antibody–drug conjugate for treatment of nectin-4–expressing cancers. Mol. Cancer Ther..

[111] Zhang J., Liu R., Gao S., Yang H., Chen J., Yuan F., Liu J., Guo H., Zhang S., Li X. (2024). 9MW2821, a nectin-4 antibody–drug conjugate (ADC), in patients with advanced solid tumor: Results from a phase 1/2a study. J. Clin. Oncol..

[112] Jain N., Smith S.W., Ghone S., Tomczuk B. (2015). Current ADC linker chemistry. Pharm. Res..

[113] Bechtold-Peters K., Ruggiero A., Vriezen N., Ihle N., Klein A., Morgan C., Schweizer D., Liu D., Jacobson F., Buecheler J. (2023). CMC regulatory considerations for antibody–drug conjugates. J. Pharm. Sci..

[114] Tao J., Gu Y., Zhou W., Wang Y. (2025). Dual-payload antibody–drug conjugates: Taking a dual shot. Eur. J. Med. Chem..

[115] Wen M., Yu A., Park Y., Calarese D., Gerber H.P., Yin G. (2025). Homogeneous antibody–drug conjugates with dual payloads: Potential, methods and considerations. mAbs.

[116] Yamazaki C.M., Yamaguchi A., Anami Y., Xiong W., Otani Y., Lee J., Ueno N.T., Zhang N., An Z., Tsuchikama K. (2021). Antibody–drug conjugates with dual payloads for combating breast tumor heterogeneity and drug resistance. Nat. Commun..

[117] Nervig C.S., Owen S.C. (2023). Advances in the development of dual-drug antibody drug conjugates. ADC Rev. J. Antib. Drug Conjug..

[118] Gu Y., Wang Z., Wang Y. (2024). Bispecific antibody drug conjugates: Making 1+1>2. Acta Pharm. Sin. B.

[119] Ji X., Yang Y., Ma C., Huang W., Guo S., Wang L., Zheng H., Wu X. (2026). Recent advances in bispecific antibody–drug conjugates for breast cancer therapy. Cancer Chemother. Pharmacol..

[120] Zhou M., Huang Z., Ma Z., Chen J., Lin S., Yang X., Gong Q., Braunstein Z., Wei Y., Rao X. (2025). The next frontier in antibody–drug conjugates: Challenges and opportunities in cancer and autoimmune therapy. Cancer Drug Resist..

[121] Ma Y., Huang Y., Zhao Y., Zhao S., Xue J., Yang Y., Fang W., Guo Y., Han Y., Yang K. (2024). BL-B01D1, a first-in-class EGFR–HER3 bispecific antibody–drug conjugate, in patients with locally advanced or metastatic solid tumours: A first-in-human, open-label, multicentre, phase 1 study. Lancet Oncol..

[122] Liu J., Burris H., Wang J.S., Barroilhet L., Gutierrez M., Wang Y., Vaze A., Commerford R., Royer-Joo S., Choeurng V. (2021). An open-label phase I dose-escalation study of the safety and pharmacokinetics of DMUC4064A in patients with platinum-resistant ovarian cancer. Gynecol. Oncol..

[123] ClinicalTrials.gov A Study Evaluating the Safety and Pharmacokinetics of DMUC4064A in Participants with Platinum-Resistant Ovarian Cancer or Unresectable Pancreatic Cancer. Identifier: NCT02146313. NCT02146313.

[124] Carneiro B.A., Papadopoulos K.P., Strickler J.H., Lassman A.B., Waqar S.N., Chae Y.K., Patel J.D., Shacham-Shmueli E., Kelly K., Khasraw M. (2023). Phase I study of anti–epidermal growth factor receptor antibody–drug conjugate serclutamab talirine: Safety, pharmacokinetics, and antitumor activity in advanced glioblastoma. Neurooncol. Adv..

[125] ClinicalTrials.gov Safety, Pharmacokinetics, and Preliminary Efficacy of BYON4413 in Acute Myeloid Leukemia and Myelodysplastic Neoplasms. Identifier: NCT06359002. NCT06359002.

[126] Daver N.G., Montesinos P., DeAngelo D.J., Wang E.S., Todisco E., Tarella C., Martinelli G., Erba H.P., Deconinck E., Sweet K.L. (2020). A phase I/II study of IMGN632, a novel CD123-targeting antibody–drug conjugate, in patients with relapsed/refractory acute myeloid leukemia, blastic plasmacytoid dendritic cell neoplasm, and other CD123-positive hematologic malignancies. J. Clin. Oncol..

[127] Cho S., Zammarchi F., Williams D.G., Havenith C.E.G., Monks N.R., Tyrer P., D’Hooge F., Fleming R., Vashisht K., Dimasi N. (2018). Antitumor activity of MEDI3726 (ADCT-401), a pyrrolobenzodiazepine antibody–drug conjugate targeting PSMA, in preclinical models of prostate cancer. Mol. Cancer Ther..

[128] Wiedemeyer W.R., Gavrilyuk J., Schammel A., Zhao X., Sarvaiya H., Pysz M., Gu C., You M., Isse K., Sullivan T. (2022). ABBV-011, a novel, calicheamicin-based antibody–drug conjugate, targets SEZ6 to eradicate small cell lung cancer tumors. Mol. Cancer Ther..

[129] Fong J.Y., Phuna Z., Chong D.Y., Heryanto C.M., Low Y.S., Oh K.C., Lee Y.H., Ng A.W.R., In L.L.A., Teo M.Y.M. (2025). Advancements in antibody–drug conjugates as cancer therapeutics. J. Natl. Cancer Cent..

[130] Song Y., Zhang J., Zhou K., Zhang L., Barve M., Lemech C., Li W., Cherng H.J.J., Huang H., Xie L. (2024). Safety and efficacy in patients with advanced lymphomas from a global phase 1a/1b, first-in-human study of CS5001, a novel anti-ROR1 ADC. Blood.

[131] Song M.M., Tolcher A.W., Gutierrez M.E., Zsiros E., Fu S., Liu J.F., Morgensztern D., Kordahi S., Nietubicz C., Barbu E.A. (2025). A phase 1 dose escalation and dose expansion study of LNCB74, a B7-H4 targeted antibody drug conjugate, as monotherapy in participants with advanced solid tumors. J. Clin. Oncol..

[132] Li Q., Cheng Y., Tong Z., Liu Y., Wang X., Yan M., Chang J., Wang S., Du C., Li L. (2024). HER2-targeting antibody drug conjugate FS-1502 in HER2-expressing metastatic breast cancer: A phase 1a/1b trial. Nat. Commun..

[133] Hu X., Zhang Q., Wang L., Zhang J., Ouyang Q., Wang X., Li W., Xie W., Tong Z., Wang S. (2025). ACE-Breast-02: A randomized phase III trial of ARX788 versus lapatinib plus capecitabine for HER2-positive advanced breast cancer. Signal Transduct. Target. Ther..

[134] Lentz R.W., Ng M.C.H., Yong W.P., Meric-Bernstam F., Singh I., Srirangam V., Cometa J., Blanchard S., Nellore R., Shah K.J. (2025). Clinical activity of EBC-129, a first-in-class, anti N256-glycosylated CEACAM5 and CEACAM6 antibody–drug conjugate (ADC), in patients with pancreatic ductal adenocarcinoma (PDAC) in a phase 1 study. J. Clin. Oncol..

[135] Skidmore L.K., Mills D., Kim J.Y., Knudsen N.A., Nelson J.D., Pal M., Wang J., GC K., Gray M.J., Barkho W. (2024). Preclinical characterization of ARX517, a site-specific stable PSMA-targeted antibody–drug conjugate for the treatment of metastatic castration-resistant prostate cancer. Mol. Cancer Ther..

[136] Le Q., Tang T.T., Leonti A., Castro S., McKay C.N., Perkins L., Pardo L., Keikey D., Hylkema T., Call L. (2023). Preclinical studies targeting CD74 with STRO-001 antibody-drug conjugate in acute leukemia. Blood Adv..

[137] Brown E.F., Colombo I., Madariaga A., Kasherman L. (2025). Immunological mechanisms and antibody-drug confugates targeting B7-H3 and B7-H4 in ovarian cancer. Front. Immunol..

[138] Scribner J.A., Hav M., Summers A., Nguyen H., Conner J., Corey E., Loo D.T. (2025). MGC026, a glycan-linked exatecan-based antibody–drug conjugate (ADC) targeting B7-H3, is efficacious toward prostate cancer patient-derived xenograft. Mol. Cancer Ther..

[139] Han H.S., Kalinsky K., Abuhadra N., Giordano A., Starks D., Wulf G.M., McAndrew N.P., O’Shaughnessy J., Spira A.I., Chan N. (2025). Emiltatug ledadotin (Emi-Le), a B7-H4–directed dolasynthen antibody–drug conjugate (ADC) being investigated in phase 1 dose expansion in patients with triple negative breast cancer who received at least one prior topoisomerase I inhibitor ADCs. J. Clin. Oncol..

[140] Hamilton E.P., Chaudhry A., Spira A.I., Adams S., Abuhadra N., Giordano A., Parajuli R., Han H.S., Weise A.M., Marchesani A. (2023). XMT-1660: A phase 1b trial of a B7-H4 targeted antibody–drug conjugate (ADC), in breast, endometrial, and ovarian cancers. J. Clin. Oncol..

[141] Zhang Z., Liang X., Huang Y., Yang L., Jiang H., Qin Y., Liu R., Gao S., Chen J., Lu C. (2025). Results from a phase 1/2 study of 7MW3711, a novel B7-H3 antibody–drug conjugate (ADC) incorporating a topoisomerase I inhibitor in patients with advanced solid tumors. J. Clin. Oncol..

[142] Li Z., Wang Q., Han L., Ouyang W., Li X., Yao Y., Sun L., Shi H., Lu S., Wang S. (2025). Results from a phase 1/2 study of 7MW3711, a novel B7-H3 antibody–drug conjugate (ADC) incorporating a topoisomerase I inhibitor in patients with lung cancer. J. Clin. Oncol..

