# Supplementary material for: ADC Conjugation Strategies: From Technological Evolution to a Practical Selection Framework
Source: Pharmaceutics. 2026 Jul 13;18(7):852. doi: 10.3390/pharmaceutics18070852 (PMC13414643; doi:10.3390/pharmaceutics18070852)
Supplement: Supplementary file 1 [file pharmaceutics-18-00852-s001.zip › pharmaceutics-4380070-supplementary.pdf]

Table S1. Complete Program-Level Listing of ADCs Using Second-Generation Conjugation Platforms.

2nd Generation Conjugation Platform (Conjugation method)	Product Name	Target	Payload Class	Indications	Manufacturer	Clinical Phase / Registry Status	Ref
THIOMAB (Engineered cysteine conjugation)	DMUC4064A	MUC16	Microtubule inhibitor (MMAE)	PROC or UPC	Genentech.	Phase I Completed (NCT02146313)	[1,2]
	DCDS0780A	CD79b	Microtubule inhibitor (MMAE)	R/R B-NHL	Hoffmann-La Roche	Phase I Completed (NCT02453087)	[3,4]
	HDP-101	BCMA	RNA polymerase II inhibitor (Amanitin)	PCD incl. MM	Heidelberg Pharma	Phase I/II Recruiting (NCT04879043)	[5]
	ABBV-321	EGFR	DNA-crosslinking agent (SGD-1882)	EGFR+AST	AbbVie	Phase I Completed (NCT03234712)	[6]
	MEDI2228	BCMA	DNA-crosslinking agent (SG3199)	R/R MM	MedImmune	Phase I Completed (NCT03489525)	[7]
	BYON3521	c-Met	DNA-alkylating agent (DUBA)	c-Met+LA/mAST	Byondis	Phase I Completed (NCT05323045)	[8]
	BYON-4413	CD123	DNA-alkylating agent (DUBA)	R/R AML or MDS	Byondis	Phase I Not recruiting (NCT06359002)	[9]
	IMGN632	CD123	DNA-alkylating agent (DGN549)	CD123+AML	AbbVie	Phase I/II Not recruiting (NCT03386513)	[10]
	ADCT-401	PSMA	DNA-crosslinking agent (SG3199)	mCRPC	MedImmune	Phase I Completed (NCT02991911)	[11]
	ABBV-011	SEZ6	DNA-cleaving agent (Calicheamicin)	R/R SCLC	AbbVie	Phase I Completed (NCT03639194)	[12]
	PYX-201	ED-B	DNA-alkylating agent (PF-06380101)	AST	Pyxis Oncology	Phase I Recruiting (NCT05720117)	[13,14]

Table S1. (Continued).

2nd Generation Conjugation Platform (Conjugation method)	Product Name	Target	Payload Class	Indications	Manufacturer	Clinical Phase / Registry Status	Ref
ConjuALL (CAAX/farnesyl transferase-mediated conjugation)	LCB84	Trop-2	Microtubule inhibitor (MMAE)	AST	LigaChem Biosciences	Phase I/II Recruiting (NCT05941507)	[15]
	CS5001	ROR1	DNA-Damaging agent (PBD dimer)	AST; Lymphomas	CStone Pharmaceuticals	Phase I Recruiting (NCT05279300)	[16]
	LNCB74	B7-H4	Microtubule inhibitor (MMAE)	AST	NextCure	Phase I Recruiting (NCT06774963)	[17]
	FS-1502	HER2	Microtubule inhibitor (MMAF)	HER2+mBC	LigaChem Biosciences	Phase I Completed (NCT03944499)	[18,19]
EuCODE (Unnatural amino acid incorporation)	ARX788	HER2	Microtubule inhibitor (AS269)	HER2+BC<br>HER2+BC (Post–T-DXd)<br>HER2+AST	Ambrx Biopharma	Phase II Not recruiting (NCT04829604)<br>Phase II/III Not recruiting (NCT05426486)<br>Phase I Completed (NCT03255070)	[18,20, 21]
	EBC-129	CEACAM5/6	Microtubule inhibitor (MMAE)	AST	Experimental Drug Development Centre (EDDC), A*STAR	Phase I Recruiting (NCT05701527)	[22]
	ARX517	PSMA	Microtubule inhibitor (AS269)	mCRPC	Janssen Research & Development	Phase I Not recruiting (NCT04662580)	[23]

Table S1. (Continued).

2nd Generation Conjugation Platform (Conjugation method)	Product Name	Target	Payload Class	Indications	Manufacturer	Clinical Phase / Registry Status	Ref
Xpress CF+ (cell-free unnatural amino acid incorporation)	STRO-001	CD74	Microtubule inhibitor (Maytansinoid)	Advanced BCMs	Sutro Biopharma	Phase I Completed (NCT03424603)	[24]
	STRO-002	FRα	Microtubule inhibitor (SC209)	Ovarian & Endometrial Cancers	Sutro Biopharma	Phase I Completed (NCT03748186)	[25]
SMAC (Sortase A-mediated conjugation)	NBE002	ROR1	Topoisomerase II inhibitor (PNU-159682)	AST	NBE-Therapeutics	Phase I/II Terminated (NCT04441099)	[26]
SMARTag (formylglycine-generating enzyme (FGE)-mediated aldehyde-tag conjugation)	TRPH-222	CD22	Microtubule inhibitor (Maytansinoid)	R/R BCL	Triphase Research and Development	Phase I Completed (NCT03682796)	[27]
mTG (microbial transglutaminase-mediated conjugation)	DP303c	HER2	Microtubule inhibitor (MMAE)	Advanced EOC	CSPC ZhongQi Pharmaceutical Technology	Phase II Not yet recruiting (NCT04828616)	[28]
				Advanced or HER2+mGC		Phase II Not yet recruiting (NCT04826107)	
				HER2+AST		Phase I Not yet recruiting (NCT04146610)	[18,28]
				HER2+BC		Phase II Not yet recruiting (NCT05334810)	[28]

Abbreviations: AML: acute myeloid leukemia; AST: advanced solid tumors; BC: breast cancer; BCMA: B-cell maturation antigen; BCMs: B-cell malignancies; BCL: B-cell lymphomas; B-NHL: B-cell non-Hodgkin lymphoma; EOC: epithelial ovarian cancer; FRα: Folate Receptor Alpha; HER2: human epidermal growth factor receptor 2; LA/mAST: locally advanced or metastatic solid tumors; MDS: myelodysplastic syndromes; mGC: metastatic gastric cancer; MM: multiple myeloma; MMAE: Monomethyl auristatin E; mBC: metastatic breast cancer; mCRPC: metastatic castration-resistant prostate cancer; PCD: plasma cell disorders; PROC: platinum-resistant ovarian cancer; ROR1: Receptor tyrosine kinase-like Orphan Receptor 1; R/R: relapsed/refractory; SCLC: small cell lung cancer; T-DXd: trastuzumab deruxtecan; Trop-2: Tumor-associated calcium signal transducer 2; UPC: unresectable pancreatic cancer. **Note:** Clinical phase and registry status were verified using ClinicalTrials.gov as of 2 July 2026. Registry status refers to the listed study and does not necessarily indicate continuation or discontinuation of the overall development program. "Completed" and "Not recruiting" should not be interpreted as "Terminated" unless explicitly designated as such. Approved ADCs are summarized separately in Table 1. Where mature clinical outcome data were not publicly available, this limitation is noted in the accompanying text.

Table S2. Complete Program-Level Listing of ADCs Using Third-Generation Conjugation Strategies.

3rd Generation Conjugation Platform (Conjugation method)	Product Name	Target	Payload Class	Indications	Manufacturer	Clinical Phase / Registry Status	Ref
GlycoConnect (Fc glycan remodeling)	IBI343	CLDN18.2	Topoisomerase I inhibitor (Exatecan)	GAC, GEJAC	Innovent	Phase III Recruiting (NCT06238843)	[299]
	IBI343	CLDN18.2	Topoisomerase I inhibitor (Exatecan)	PDAC	Innovent	Phase II Recruiting (NCT06770439)	[30,31]
	MRG004A	Tissue Factor	Microtubule inhibitor (MMAE)	PC, TNBC	Lepu Biopharma	Phase I/II Completed (NCT04843709)	[32,33]
	MGC026	B7-H3	Topoisomerase I inhibitor (Exatecan derivative)	CRPC	MacroGenics	Phase I Recruiting (NCT06242470)	[34,35]
	Emi-Le (XMT-1660)	B7-H4	Microtubule inhibitor (AF-HPA)	BC, EC, OC	Mersana Therapeutics	Phase I Recruiting (NCT05377996)	[36,37]
AbClick (proximity-induced Lys248 conjugation)	AT-211 (DA-3501)	CLDN18.2	Microtubule inhibitor (MMAE)	GAC, GEJAC, PDAC	AbTis / Dong-A ST	Phase I/II Recruiting (NCT07481357)	[388]
ThioBridge (disulfide rebridging)	MBRC-101	EphA5	Microtubule inhibitor (MMAE)	cUADT, cGU, BC	MBrace Therapeutics	Phase I/II Recruiting (NCT06014658)	[39]
IDconnect (disulfide rebridging)	9MW2821	Nectin-4	Microtubule inhibitor (MMAE)	mUC, mCC	Mabwell Bioscience	Phase III Recruiting (NCT06592326) Phase III Recruiting (NCT06692166)	[40]
	7MW3711	B7-H3	Topoisomerase I inhibitor (Exatecan derivative)	ESC, OC, LC	Mabwell Bioscience	Phase I/II Recruiting (NCT06008366) Phase I/II Recruiting (NCT06008379)	[41,42]

Abbreviations: AF-HPA: auristatin F hydroxypropylamide; BC: breast cancer; mCC: Metastatic Cervical Cancer; CLDN: Claudin; CRPC: castration-resistant prostate cancer; cUADT: cancer of upper aerodigestive tract; EC: endometrial cancer; ESC: esophageal cancer; EphA: Ephrin type-A receptor; GAC: gastric adenocarcinoma; GEJAC: gastroesophageal junction adenocarcinoma; cGU: cancer of genitourinary; LC: lung cancer; MMAE: Monomethyl auristatin E; mUC: metastatic urothelial carcinoma; OC: ovarian cancer; PC: pancreatic cancer; PDAC: pancreatic ductal adenocarcinoma; TNBC: triple-negative breast cancer. Note: Clinical

phase and registry status were verified using ClinicalTrials.gov as of 2 July 2026. Registry status refers to the listed study and does not necessarily indicate continuation or discontinuation of the overall development program. “Completed” and ″Not recruiting” should not be interpreted as “Terminated” unless explicitly designated as such. Approved ADCs are summarized separately in Table 1. Where mature clinical outcome data were not publicly available, this limitation is noted in the accompanying text.

References

1. Liu, J.; Burris, H.; Wang, J.S.; Barroilhet, L.; Gutierrez, M.; Wang, Y.; Vaze, A.; Commerford, R.; Royer-Joo, S.; Choeurng, V.; et al. An open-label phase I dose-escalation study of the safety and pharmacokinetics of DMUC4064A in patients with platinum-resistant ovarian cancer. *Gynecol. Oncol.* **2021**, *163*, 473–480. https://doi.org/10.1016/j.ygyno.2021.09.023

2. ClinicalTrials.gov. A Study Evaluating the Safety and Pharmacokinetics of DMUC4064A in Participants With Platinum-Resistant Ovarian Cancer or Unresectable Pancreatic Cancer. Identifier: NCT02146313. Available online: https://clinicaltrials.gov/study/NCT02146313 (accessed on 2 July 2026).

3. ClinicalTrials.gov. A Study of Escalating Doses of DCDS0780A in Participants with B-Cell Non-Hodgkin Lymphoma. Identifier: NCT02453087. Available online: https://clinicaltrials.gov/study/NCT02453087 (accessed on 2 July 2026).

4. Herrera, A.F.; Patel, M.R.; Burke, J.M.; Advani, R.; Cheson, B.D.; Sharman, J.P.; Penuel, E.; Polson, A.G.; Liao, C.D.; Li, C.; et al. Anti-CD79B antibody–drug conjugate DCDS0780A in patients with B-cell non-Hodgkin lymphoma: phase 1 dose-escalation study. *Clin. Cancer Res.* **2022**, *28*, 1294–1301. https://doi.org/10.1158/1078-0432.CCR-21-3261

5. Orlowski, R.Z.; Richard, S.; Kaufman, J.L.; et al. The anti-BCMA antibody–drug conjugate HDP-101 with a novel amanitin payload shows promising initial first-in-human results in relapsed multiple myeloma. *Blood*. **2024**, 144, 3381. https://doi.org/10.1182/blood-2024-210088

6. Carneiro, B.A.; Papadopoulos, K.P.; Strickler, J.H.; Lassman, A.B.; Waqar, S.N.; Chae, Y.K.; Patel, J.D.; Shacham-Shmueli, E.; Kelly, K.; Khasraw, M.; et al. Phase I study of anti–epidermal growth factor receptor antibody–drug conjugate serclutamab talirine: safety, pharmacokinetics, and antitumor activity in advanced glioblastoma. *Neurooncol. Adv.* **2023**, *5*, vdac183. https://doi.org/10.1093/noajnl/vdac183

7. Dimopoulos, M.A.; Migkou, M.; Bhutani, M.; Ailawadhi, S.; Kalff, A.; Walcott, F.L.; Pore, N.; Brown, M.; Wang, F.; Cheng, L.I.; et al. Phase 1 first-in-human study of MEDI2228, a BCMA-targeted ADC, in patients with relapsed refractory multiple myeloma. Leuk. *Lymphoma.* **2024**, *65*, 1789–1800. https://doi.org/10.1080/10428194.2024.2373331

8. Kotecki, N.; van Herpen, C.M.L.; Curigliano, C.; Hendriks, M.; Vermaas, T.C.; Corrigan, L.; et al. Abstract CT185: First-in-human dose-escalation trial with the c-MET-targeting antibody–drug conjugate BYON3521. *Cancer Research.* **2023**, 83, CT185. https://doi.org/10.1158/1538-7445.AM2023-CT185

9. ClinicalTrials.gov. Safety, Pharmacokinetics, and Preliminary Efficacy of BYON4413 in Acute Myeloid Leukemia and Myelodysplastic Neoplasms. Identifier: NCT06359002. Available online: https://clinicaltrials.gov/study/NCT06359002 (accessed on 2 July 2026).

10. Daver, N.G.; Montesinos, P.; DeAngelo, D.J.; Wang, E.S.; Todisco, E.; Tarella, C.; Martinelli, G.; Erba, H.P.; Deconinck, E.; Sweet, K.L.; et al. A phase I/II study of IMGN632, a novel CD123-targeting antibody–drug conjugate, in patients with relapsed/refractory acute myeloid leukemia, blastic plasmacytoid dendritic cell neoplasm, and other CD123-positive hematologic malignancies. *J. Clin. Oncol.* **2020**, *38*, TPS7563. https://doi.org/10.1200/JCO.2020.38.15_suppl.TPS7563

11. Cho, S.; Zammarchi, F.; Williams, D.G.; Havenith, C.E.G.; Monks, N.R.; Tyrer, P.; D'Hooge, F.; Fleming, R.; Vashisht, K.; Dimasi, N.; et al. Antitumor activity of MEDI3726 (ADCT-401), a pyrrolobenzodiazepine antibody–drug conjugate targeting PSMA, in preclinical models of prostate cancer. *Mol. Cancer Ther.* **2018**, *17*, 2176–2186. https://doi.org/10.1158/1535-7163.MCT-17-0982

12. Wiedemeyer, W.R.; Gavrilyuk, J.; Schammel, A.; Zhao, X.; Sarvaiya, H.; Pysz, M.; Gu, C.; You, M.; Isse, K.; Sullivan, T.; et al. ABBV-011, a novel, calicheamicin-based antibody–drug conjugate, targets SEZ6 to eradicate small cell lung cancer tumors. *Mol. Cancer Ther.* **2022**, *21*, 986–998. https://doi.org/10.1158/1535-7163.MCT-21-0851

13. Fan, Q.; Chen, H.; Wei, G.; Wei, D.; Wang, Z.; Zhang, L.; Wang, J.; Zhu, M. A review of conjugation technologies for antibody–drug conjugates. *Antib. Ther.* **2025**, *8*, 157–170. https://doi.org/10.1093/abt/tbaf010

14. Fong, J.Y.; Phuna, Z.; Chong, D.Y.; Heryanto, C.M.; Low, Y.S.; Oh, K.C.; Lee, Y.H.; Ng, A.W.R.; In, L.L.A.; Teo, M.Y.M. Advancements in antibody–drug conjugates as cancer therapeutics. *J. Natl. Cancer Cent.* **2025**, *5*, 362–378. https://doi.org/10.1016/j.jncc.2025.01.007

15. Liu, X.; Ma, L.; Li, J.; Sun, L.; Yang, Y.; Liu, T.; Xing, D.; Yan, S.; Zhang, M. Trop2-targeted therapies in solid tumors: advances and future directions. *Theranostics.* **2024**, *14*, 3674–3692. https://doi.org/10.7150/thno.98178

16. Song, Y.; Zhang, J.; Zhou, K.; Zhang, L.; Barve, M.; Lemech, C.; Li, W.; Cherng, H.J.J.; Huang, H.; Xie, L.; et al. Safety and efficacy in patients with advanced lymphomas from a global phase 1a/1b, first-in-human study of CS5001, a novel anti-ROR1 ADC. *Blood.* **2024**, *144*, 1739. https://doi.org/10.1182/blood-2024-192979

17. Song, M.M.; Tolcher, A.W.; Gutierrez, M.E.; Zsiros, E.; Fu, S.; Liu, J.F.; Morgensztern, D.; Kordahi, S.; Nietubicz, C.; Barbu, E.A.; et al. A phase 1 dose escalation and dose expansion study of LNCB74, a B7-H4 targeted antibody drug conjugate, as monotherapy in participants with advanced solid tumors. *J. Clin. Oncol.* **2025**, *43*, TPS3167. https://doi.org/10.1200/JCO.2025.43.16_suppl.TPS3167

18. Najminejad, Z.; Dehghani, F.; Mirzaei, Y.; Mer, A.H.; Saghi, S.A.; Abdolvahab, M.H.; Bagheri, N.; Meyfour, A.; Jafari, A.; Jahandideh, S.; et al. Clinical perspective: antibody–drug conjugates for the treatment of HER2-positive breast cancer. *Mol. Ther.* **2023**, *31*, 1874–1903. https://doi.org/10.1016/j.ymthe.2023.03.019

19. Li, Q.; Cheng, Y.; Tong, Z.; Liu, Y.; Wang, X.; Yan, M.; Chang, J.; Wang, S.; Du, C.; Li, L.; et al. HER2-targeting antibody drug conjugate FS-1502 in HER2-expressing metastatic breast cancer: a phase 1a/1b trial. *Nat. Commun.* **2024**, *15*, 5158. https://doi.org/10.1038/s41467-024-48798-w

20. Niu, N.; Xue, J.; Chen, G.; Qiu, F.; Xu, Q.; Zheng, X.; Liu, C.; Zhao, Y.; Gu, X.; Zhao, Y.; et al. Neoadjuvant ARX788 plus pyrotinib versus trastuzumab, pertuzumab, docetaxel and carboplatin for HER2-positive breast cancer: a randomized phase 2b trial. *Nat. Commun.* **2025**, *16*, 6036. https://doi.org/10.1038/s41467-025-61213-2

21. Hu, X.; Zhang, Q.; Wang, L.; Zhang, J.; Ouyang, Q.; Wang, X.; Li, W.; Xie, W.; Tong, Z.; Wang, S.; et al. ACE-Breast-02: a randomized phase III trial of ARX788 versus lapatinib plus capecitabine for HER2-positive advanced breast cancer. *Signal Transduct. Target. Ther.* **2025**, *10*, 56. https://doi.org/10.1038/s41392-025-02149-3

22. Lentz, R.W.; Ng, M.C.H.; Yong, W.P.; Meric-Bernstam, F.; Singh, I.; Srirangam, V.; Cometa, J.; Blanchard, S.; Nellore, R.; Shah, K.J.; et al. Clinical activity of EBC-129, a first-in-class, anti N256-glycosylated CEACAM5 and CEACAM6 antibody–drug conjugate (ADC), in patients with pancreatic ductal adenocarcinoma (PDAC) in a phase 1 study. *J. Clin. Oncol.* **2025**, *43*, 4018. https://doi.org/10.1200/JCO.2025.43.16_suppl.4018

23. Skidmore, L.K.; Mills, D.; Kim, J.Y.; Knudsen, N.A.; Nelson, J.D.; Pal, M.; Wang, J.; GC, K.; Gray, M.J.; Barkho, W.; et al. Preclinical characterization of ARX517, a site-specific stable PSMA-targeted antibody–drug conjugate for the treatment of metastatic castration-resistant prostate cancer. *Mol. Cancer Ther.* **2024**, *23*, 1842–1853. https://doi.org/10.1158/1535-7163.MCT-23-0927

24. Le, Q.; Tang, T.T.; Leonti, A.; Castro, S.; McKay, C.N.; Perkins, L.; Pardo, L.; Keikey, D.; Hylkema, T.; Call, L.; et al. Preclinical studies targeting CD74 with STRO-001 antibody-drug conjugate in acute leukemia. *Blood Adv.* **2023**, *7*, 1666–1670. https://doi.org/10.1182/bloodadvances.2022008303

25. Li, X.; Zhou, S.; Abrahams, C.L.; Krimm, S.; Smith, J.; Bajjuri, K.; Stephenson, H.T.; Henningsen, R.; Hanson, J.; Heibeck, T.H.; et al. Discovery of STRO-002, a novel homogeneous ADC targeting folate receptor alpha for the treatment of ovarian and endometrial cancers. *Mol. Cancer Ther.* **2023**, *22*, 155–167. https://doi.org/10.1158/1535-7163.MCT-22-0322

26. ClinicalTrials.gov. NBE-002 in Patients With Advanced Solid Tumors. Identifier: NCT04441099. Available online: https://clinicaltrials.gov/study/NCT04441099 (accessed on 2 July 2026).

27. Hernandez-Ilizaliturri, F.J.; Flinn, I.W.; Kuruvilla, J.; Assouline, S.E.; Ulrickson, M.L.; Christian, B.A.; Landsburg, D.J.; Stuart, M.; Lowman, H.; Levin, N. A phase I pharmacokinetic (PK) and safety study of Trph-222 in patients with relapsed/refractory B-cell non-Hodgkin lymphoma (R/R NHL): dose-escalation results. *Blood.* **2020**, *136*, 41–42. https://doi.org/10.1182/blood-2020-142859

28. Zhang, J.; Du, Y.; Meng, Y.; Liu, X.; Mu, Y.; Liu, Y.; Shi, Y.; Wang, J.; Zang, A.; Gu, S.; et al. First-in-human study of DP303c, a HER2-targeted antibody-drug conjugate in patients with HER2 positive solid tumors. *NPJ Precis. Oncol.* 2024, *8*, 200. https://doi.org/10.1038/s41698-024-00687-7

29. Liu, J.; Yang, J.; Sun, Y.; Gong, J.; Yue, J.; Pan, Y.; Sun, M.; Song, R.; Xiao, X.; Tazbirkova, A.; et al. CLDN18.2-targeting antibody–drug conjugate IBI343 in advanced gastric or gastroesophageal junction adenocarcinoma: a phase 1 trial. *Nat. Med.* **2025**, *31*, 3028–3036. https://doi.org/10.1038/s41591-025-03783-8

30. Yu, X.; Zhang, J.; Tazbirkova, A.; Yang, J.; Yue, J.; Sun, Y.; Pan, Y.; Sun, M.; Qin, Y.; Shen, L.; et al. Safety and efficacy of IBI343 (anti-claudin18.2 antibody–drug conjugate) in patients with advanced pancreatic ductal adenocarcinoma or biliary tract cancer: preliminary results from a phase 1 study. *J. Clin. Oncol.* **2024**, *42*, 3037. https://doi.org/10.1200/JCO.2024.42.16_suppl.3037

31. Yu, X.; Zhang, J.; Liu, J.J.; Yang, J.; Yue, J.; Sun, Y.; Pan, Y.; Sun, M.L.; Qin, Y.; Shen, L.; et al. 132MO Anti-claudin18.2 (CLDN18.2) antibody–drug conjugate (ADC) IBI343 in patients with advanced pancreatic ductal adenocarcinoma (PDAC): updated results from a phase I study. *Ann. Oncol.* **2024**, *35*, S1456. https://doi.org/10.1016/j.annonc.2024.10.158

32. Park, W.; Zhang, J.; Dayyani, F.; Shan, J.; Liu, R.; Guo, R.; O'Reilly, E.M.; Liu, Z.; Gao, S.; Wu, X.; et al. Phase I/II first-in-human study to evaluate the safety and efficacy of tissue factor ADC MRG004A in patients with solid tumors. *J.*

Clin. Oncol. **2024**, *42*, 3002. https://doi.org/10.1200/JCO.2024.42.16_suppl.3002

33. Ren, W.; Park, W.; Yu, X.; Shan, J.; Wu, H.; Zhao, H.; Hu, C.; Tang, J.; Yang, J.; Niu, Z.; et al. Tissue factor (TF) antibody–drug conjugate (ADC) MRG004A in patients (pts) with advanced pancreatic cancer (PC): updated results from a phase I study. *Ann. Oncol.* **2025**, *36*, S1152. https://doi.org/10.1016/j.annonc.2025.08.2842

34. Brown, E.F.; Colombo, I.; Madariaga, A.; Kasherman, L. Immunological mechanisms and antibody-drug confugates targeting B7-H3 and B7-H4 in ovarian cancer. *Front. Immunol.*. **2025**, *16*, 2025. https://doi.org/10.3389/fimmu.2025.1683674

35. Scribner, J.A.; Hav, M.; Summers, A.; Nguyen, H.; Conner, J.; Corey, E.; Loo, D.T. MGC026, a glycan-linked exatecan-based antibody–drug conjugate (ADC) targeting B7-H3, is efficacious toward prostate cancer patient-derived xenograft. Mol. *Cancer Ther.* **2025**, *24*, A140. https://doi.org/10.1158/1535-7163.TARG-25-A140

36. Han, H.S.; Kalinsky, K.; Abuhadra, N.; Giordano, A.; Starks, D.; Wulf, G.M.; McAndrew, N.P.; O'Shaughnessy, J.; Spira, A.I.; Chan, N.; et al. Emiltatug ledadotin (Emi-Le), a B7-H4–directed dolasynthen antibody–drug conjugate (ADC) being investigated in phase 1 dose expansion in patients with triple negative breast cancer who received at least one prior topoisomerase I inhibitor ADCs. *J. Clin. Oncol.* **2025**, *43*, TPS1141. https://doi.org/10.1200/JCO.2025.43.16_suppl.TPS1141

37. Hamilton, E.P.; Chaudhry, A.; Spira, A.I.; Adams, S.; Abuhadra, N.; Giordano, A.; Parajuli, R.; Han, H.S.; Weise, A.M.; Marchesani, A.; et al. XMT-1660: A phase 1b trial of a B7-H4 targeted antibody–drug conjugate (ADC), in breast, endometrial, and ovarian cancers: *J. Clin. Oncol.* **2023**, *41*, TPS3154. https://doi.org/10.1200/JCO.2023.41.16_suppl.TPS3154

38. ClinicalTrials.gov. A First-In-Human Phase I/IIa Study to Evaluate DA 3501 in Patients with Advanced Gastric or Gastro-Esophageal Junction Adenocarcinoma and Pancreatic Ductal Adenocarcinoma. Identifier: NCT07481357. Available online: https://clinicaltrials.gov/study/NCT07481357 (accessed on 2 July 2026).

39. Sen, S.; Salkeni, M.A.; Albany, C.; Gandhi, N.J.; Edenfield, W.J.; Parsons, K.; Chen, I.; Powderly, J.D. A multi-center, open-label phase 1/1b dose finding, safety, and pharmacokinetic study of MBRC-101, an anti-EphA5 monomethyl auristatin (MMAE) antibody drug conjugate, in advanced refractory solid tumors. *J. Clin. Oncol.* **2024**, *42*, TPS3161. https://doi.org/10.1200/JCO.2024.42.16_suppl.TPS3161

40. Zhang, J.; Liu, R.; Wang, S.; Feng, Z.; Yang, H.; Gao, S.; Li, X.; Yao, X.; Chen, J.; Gong, Z.; et al. Bulumtatug fuvedotin (BFv, 9MW2821), a next-generation nectin-4–targeting antibody–drug conjugate, in patients with advanced solid tumors: a first-in-human, open-label, multicenter phase I/II study. *Ann. Oncol.* **2025**, *36*, 934–943. https://doi.org/10.1016/j.annonc.2025.04.009

41. Zhang, Z.; Liang, X.; Huang, Y.; Yang, L.; Jiang, H.; Qin, Y.; Liu, R.; Gao, S.; Chen, J.; Lu, C.; et al. Results from a phase 1/2 study of 7MW3711, a novel B7-H3 antibody–drug conjugate (ADC) incorporating a topoisomerase I inhibitor in patients with advanced solid tumors. *J. Clin. Oncol.* **2025**, *43*, 3035. https://doi.org/10.1200/JCO.2025.43.16_suppl.3035

42. Li, Z.; Wang, Q.; Han, L.; Ouyang, W.; Li, X.; Yao, Y.; Sun, L.; Shi, H.; Lu, S.; Wang, S.; et al. Results from a phase 1/2 study of 7MW3711, a novel B7-H3 antibody–drug conjugate (ADC) incorporating a topoisomerase I inhibitor in patients with lung cancer. *J. Clin. Oncol.* **2025**, *43*, 3036. https://doi.org/10.1200/JCO.2025.43.16_suppl.3036
